# Protein Adsorption Kinetics on Silica: Theoretical Modeling and Experiments

**DOI:** 10.1021/acs.langmuir.6c00098

**Published:** 2026-04-06

**Authors:** Monika Wasilewska, Agata Pomorska Gawel, Maria Morga, Małgorzata Nattich-Rak, Zbigniew Adamczyk, Boris Y. Rubinstein, Alexander M. Leshansky

**Affiliations:** † Jerzy Haber Institute of Catalysis and Surface Chemistry, 132074 Polish Academy of Sciences, Niezapominajek 8, 30−239 Krakow, Poland; ‡ Stowers Institute for Medical Research Kansas City, 1000 East 50th Street, Kansas, Missouri 64110, United States; § Department of Chemical Engineering, Technion − IIT, Haifa 32000, Israel

## Abstract

Protein adsorption mechanism on silica/electrolyte interfaces was determined experimentally and by a thorough theoretical modeling. Primarily, the adsorption kinetics of myoglobin, albumins, and fibrinogen on a silica sensor under well-defined physicochemical conditions were investigated using the quartz microbalance method. Acquired results were scaled using adsorption kinetics derived from the random sequential adsorption modeling calibrated using atomic force microscopy (AFM) analysis of protein layers. The experimental results expressed in this way were interpreted in terms of the hydrodynamic theory, considering both a rigid and a soft, lubricated contact of molecules with the surface. The theory also furnished an analytical expression enabling the calculation of adsorption efficiency (impedance) for a broad range of protein sizes. It was demonstrated that the quartz microbalance measurements interpreted in terms of theoretical impedances pertinent to the contact agreed with analogous measurements derived from the reflectometric method. This allowed to confirm a nonlocalized and monolayer adsorption mechanism of the protein molecules controlled by electrostatic interactions.

## Graphical abstract

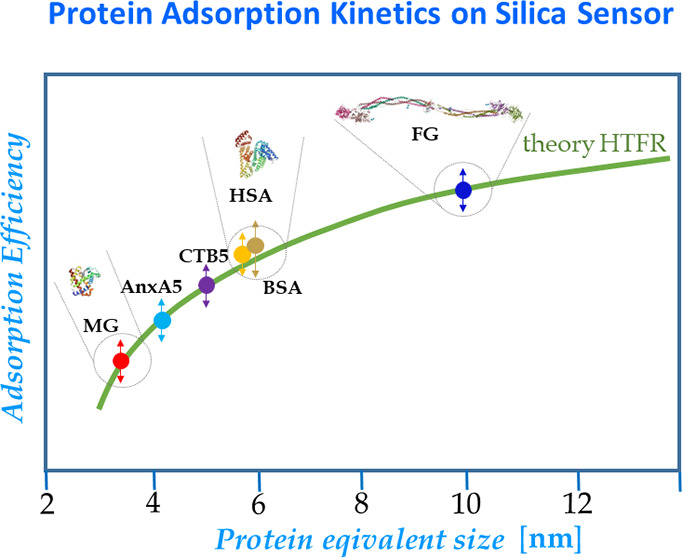

## 1 Introduction

Protein adsorption at solid/electrolyte interfaces is essential for their effective separation and purification using chromatography and filtration, as well as for applications in biosensing, bioreactors, tissue culture, and immunological assays. In medicine, the adsorption of blood serum proteins is used for controlled coating of orthopedic titanium implants, pacemakers, and catheters, which prevents the adhesion of platelets and bacteria. Conversely, unwanted adsorption of certain proteins can cause plaque formation, inflammatory responses, implant failure, sensor blockage, or fouling of ultrafiltration units.^1–3^

Because of its essential importance, protein adsorption was extensively studied using various experimental techniques. Among these, optical methods such as reflectometry and ellipsometry,^4–7^ total internal reflection fluorescence (TIRF),^6,8^ optical waveguide light-mode spectroscopy (OWLS),^9^ and surface plasmon resonance (SPR)^10–12^ were commonly used. Direct imaging by atomic force microscopy (AFM)^13–15^ enabled nanoscale characterization of adsorbed layers, while electrokinetic approaches, including streaming current and streaming potential measurements,^16–18^ were employed to determine the electrokinetic properties of adsorbed protein layers. It should be mentioned, however, that many of these methods experience shortcomings, requiring, for example, fluorescent labeling (TIRF), some are less sensitive and imprecise in the low-protein-coverage range (optical methods), whereas other methods become insensitive for the higher-coverage range (AFM, electrokinetic methods).

In a plethora of investigations, the electro-acoustic quartz crystal microbalance (QCM) method was used to study protein adsorption kinetics.^9,19–32^ This label-free method enables exceptionally sensitive real-time, in situ measurements under convective (flow) conditions for a variety of bare and functionalized sensors.^9,19–32^ However, a quantitative interpretation of the primary QCM signals (frequency and dissipation shifts) aimed at acquiring the real adsorption kinetics of proteins was not attempted because of the lack of an adequate theoretical approach. Usually, the kinetic runs derived from QCM were presented in the form of frequency and dissipation shifts vs time plots. In a few papers,^9,28–30^ the Sauerbrey equation was applied to calculate the time-dependence of the apparent coverage (mass per unit area) of protein layers using frequency shifts at different overtones. However, the Sauerbrey coverage calculated for albumin, fibrinogen and respective antibodies at the end of kinetic runs was a few times larger than the coverage determined by ellipsometry or OWLS, but this striking discrepancy was not interpreted.^9^

In some studies,^19,24^ the Voigt viscoelastic model was employed to interpret the experimental results. However, this model is physically questionable and can only yield adequate theoretical predictions for a limited range of the oscillation frequency.^21^

To obtain more precise information on protein adsorption kinetics, advanced QCM cells were developed, enabling simultaneous in situ registration of frequency shifts and optical signals for the same substrate.^20,21^

The adsorption of globular proteins on silica and gold sensors was investigated.^33–36^ The frequency shifts obtained from QCM runs were converted into respective kinetic runs using the Sauerbrey equation. These results were compared with theoretical predictions derived from the convective-diffusion equation.

In contrast to previous approaches, based on phenomenological or empirical models, in our work a quantitative interpretation of experimental results is carried out applying the *ab initio* hydrodynamic theory for discrete adsorbates and the coarse-grained Monte Carlo type modeling^37,38^ This enables derivation of closed-form impedance expressions for broad range of protein sizes valid both for a rigid contact of the molecules with the QCM sensor and for a lubricated contact applicable for a nonlocalized adsorption mechanism. The latter model is applied here for the first time to analyze protein adsorption kinetics. Subsequently, a quantitative analysis of protein adsorption/desorption kinetics is performed, providing accurate information about their adsorption mechanism and the role of electrostatic interactions. Attention is focused on proteins of key importance, such as myoglobin, albumins (human serum and bovine serum), and fibrinogen.

Myoglobin (MG) is a globular protein whose primary physiological role comprises the delivery of oxygen to tissues and the regulation of nitric oxide transport in the myocardium.^39^ The molecule consists of a single polypeptide chain of 153 amino acid residues, forming eight α-helices (around 70% of the sequence) connected by nonhelical loops.^40^ Based on its chemical composition, the molar mass of myoglobin is equal 17,800 g mol^–1^,^41^ and its density amounts to 1.35 g cm^–3^.^42^

Human serum albumin (HSA) is a nonglycosylated, single-chain protein composed of 585 amino acids, with a molar mass of 66,439 g mol^‑1^ ^43^ and a density of 1.35 g cm^–3^. Its crystalline structure is mainly formed by α-helical motifs (69%) and includes 23% β-sheet regions, stabilized by 17 intramolecular disulfide bonds.^44^ Physiologically, HSA plays an important role in maintaining osmotic pressure and mediating the transport of various ligands, including fatty acids, calcium ions, drugs, and hormones.^43–45^ BSA molecules, with a molar mass of 66,430 g mol^‑1^ and a density of 1.35 g cm^–3^, exhibit similar properties to HSA. It is extensively used in biochemical and biophysical research as a standard protein, stabilizing agent, and blocking reagent to prevent nonspecific binding in assays such as ELISA, Western blotting, and surface adsorption studies.^43^

Fibrinogen (FG) exhibiting a molar mass of 340,000 g mol^‑1^ ^44^ and a density of 1.35 g cm^–3^ plays a vital role in the hemostatic system, participating in blood clotting, wound healing, and pathological processes such as neoplasia.^46^ Structurally, fibrinogen molecule consists of two symmetrical parts, each composed of three distinct polypeptide chains: Aα, Bβ, and γ. Fibrinogen shows a strong tendency to adsorb onto various surfaces under diverse conditions,^1,35,36,47,48^ thereby mediating cellular interactions that are essential for the biocompatibility of materials.^46^ Conversely, fibrinogen layers adsorbed onto synthetic surfaces can promote platelet adhesion, often leading to fouling of artificial organs.^48^

## 2 Methods

### 2.1 Materials and Experimental Methods

Fibrinogen from human blood plasma, fractions I, type I–S in the form of crystalline powders containing 50–70% protein, 25% sodium chloride, and 15% sodium citrate; myoglobin stemming from the equine muscles supplied in the form of a lyophilized powder 95–100%, and human serum albumin (HSA) in the form of a lyophilized powder 99% having the nominal fatty acid content of 0.02% were purchased from Sigma-Aldrich. Sigma-Aldrich also supplied phosphate-buffered saline (PBS) and sodium chloride (NaCl). Sodium hydroxide and hydrochloric acid were obtained from Avantor Performance Materials Poland S.A. (formerly POCH S.A., Gliwice, Poland). All materials used in the study were analytical-grade reagents and were used without further purification.

The bulk concentration of proteins was spectrophotometrically determined after dissolving the powder in an appropriate electrolyte and after filtration, using a Shimadzu UV-2600 apparatus, exploiting the peak absorption at 409 nm for myoglobin, and 280 nm for albumin and fibrinogen.

Other chemical reagents, such as sodium chloride, hydrochloric acid, and buffers, were commercial products of Sigma-Aldrich and were used without additional purification.

Ultrapure water was obtained using the Milli-Q Elix&Simplicity 185 purification system from Millipore.

The diffusion coefficient of protein molecules was determined by dynamic light scattering (DLS) using the Zetasizer Nano ZS instrument from Malvern. The hydrodynamic diameter was calculated using the Stokes–Einstein relationship. The electrophoretic mobility of particles was measured using the Laser Doppler velocimetry (LDV) technique, which employed the same apparatus. The zeta potential was calculated using Henry’s formula.

Silica-coated AT-cut quartz crystals with a fundamental shear oscillation frequency of 5 MHz (QSense, Sweden) were used in QCM investigations. Before conducting measurements, the sensors were cleaned in a diluted piranha solution (1:1:1 ratio of deionized water, hydrogen peroxide, and sulfuric acid) for 1.5 min, thoroughly rinsed with ultrapure water, and then immersed in water at 80 °C for 30 min. Afterward, the sensors were dried in a stream of pure nitrogen and used immediately after cleaning. The QCM measurements followed the standard procedure described in refs 33–35,49 using the Q-Sense QCM instrument (Biolin Scientific, Stockholm, Sweden). First, a stable baseline in a pure electrolyte of a fixed concentration (either 1 or 10 mM NaCl, and pH 5.8) was established in the QCM-D cell at a defined flow rate (typically 2.5 × 10^–3^ cm^3^ s^–1^). Then, a protein solution of appropriate concentration was flushed through the cell until a stable signal was recorded. After a set period, the desorption phase was initiated, during which a pure electrolyte solution of the same pH and ionic strength was flushed through the cell.

The topography of the sensors was determined by atomic force microscopy (AFM) imaging under ambient conditions in semicontact mode using the NT-MDT Solver BIO device with the SMENA SFC050L scanning head. In this way, relevant parameters were determined (Supporting Information). The root-mean-square (RMS) of the sensors was 0.90 ± 0.1 nm; the roughness correlation length and wavelength were 70 ± 10 and 110 ± 10 nm, respectively. The AFM method was also used to determine adsorbed protein coverage via a direct enumeration procedure applied before in refs 36,50 . These results were used to determine the mass transfer coefficients required in the random sequential adsorption modeling.

Protein adsorption measurements were also carried out using optical reflectometry in a microfluidic impinging–jet cell, according to the previously described procedure.^51^ The adsorbents were oxidized silicon plates, carefully cleaned before each experiment using a piranha solution (H_2_SO_4_/H_2_O_2_ 1:1) and ultrapure water. The fixed-angle reflectometer was equipped with a polarized green diode laser operating at a wavelength of 532 nm (World Star Tech TECGL–532 Series, Canada). The dry mass of the adsorbates was calculated from the reflectometry signal using a homogeneous slab model.^52,53^ The root-mean-square (*rms*) of the plates was equal to 0.40 ± 0.05 nm.

The Optical Waveguide Lightmode Spectroscopy (OWLS) measurements were performed using the OWLS 210 instrument (Microvacuum Ltd., Budapest, Hungary), equipped with a laminar slit shear flow cell.^54^ The substrates were planar optical waveguides (OW 2400 from MicroVacuum, Budapest, Hungary) made of glass (refractive index *n*_S_ = 1.526) and covered with a 170 nm-thick film of Si0.78Ti0.22O2 with a refractive index *n*_F_ = 1.8. The sensor surface was coated with an additional layer (10 nm) of pure SiO_2_. A diffractive grating on the surface of the waveguide incouples He–Ne laser light at two well-defined incident angles for the transverse electric (TE) and magnetic (TM) polarization modes.^54^ Different models described in the Supporting Information were used to calculate the mass of adsorbed protein.

All experiments have been performed at 298 K.

### 2.2 Theoretical Methods

The hydrodynamic problem of the flow and the viscous stress due to a spherical particle placed in an incompressible liquid above a resonator (sensor) undergoing small-amplitude horizontal oscillations resulting in the surface velocity **υ** = υ_0_ *x̂* cos­(*ωt*), where with υ_0_ and ω are the amplitude and the frequency of oscillations, respectively was considered in refs 38,55,56 . For a freely suspended particle of the radius *a*, maintaining a minimal vertical clearance *h* from the resonator, the problem was studied theoretically for moderate separation distances (*h* ≳ 0.25*a*) in ref 55. For a small freely suspended particle at close proximity (*h* ≪ *a* ≪ δ) the analogous problem was analyzed by Sadowska et al.;^56^ here $\delta =\sqrt{2\nu /\omega }$ is the viscous penetration depth and ν = η/ρ the liquid kinematic viscosity, η is the dynamic viscosity and ρ is the density. A closely related problem of an adsorbed particle (*h* ≈ 0) with rigid contact was investigated by Leshansky et al.^38^

Physically, under the adsorbed regime, the particle is assumed to be firmly attached to the resonator oscillating in phase with it without rotation. In this case, the hydrodynamic force exerted on the particle is transmitted to the resonator via an unspecified contact force. On the other hand, under the suspended regime the particle maintains a fixed vertical clearance from the sensor, while it can execute rotary and translatory oscillatory motions characterized by a different phase and amplitude as compared to the resonator. In the latter case, the change in the force applied to the resonator is due to the modified hydrodynamic stress owing to the particle presence.

The numerically computed perturbed flow field (streamlines) and pressure due to a freely suspended particle of scaled radius *a*/δ = 0.03 and the dimensionless minimal separation distance ϵ = *h*/*a* = 0.12 are presented in Figure 1. Analogous results obtained for a rigidly adsorbed particle are shown in the Supporting Information (Figure S1),

**Figure 1:**
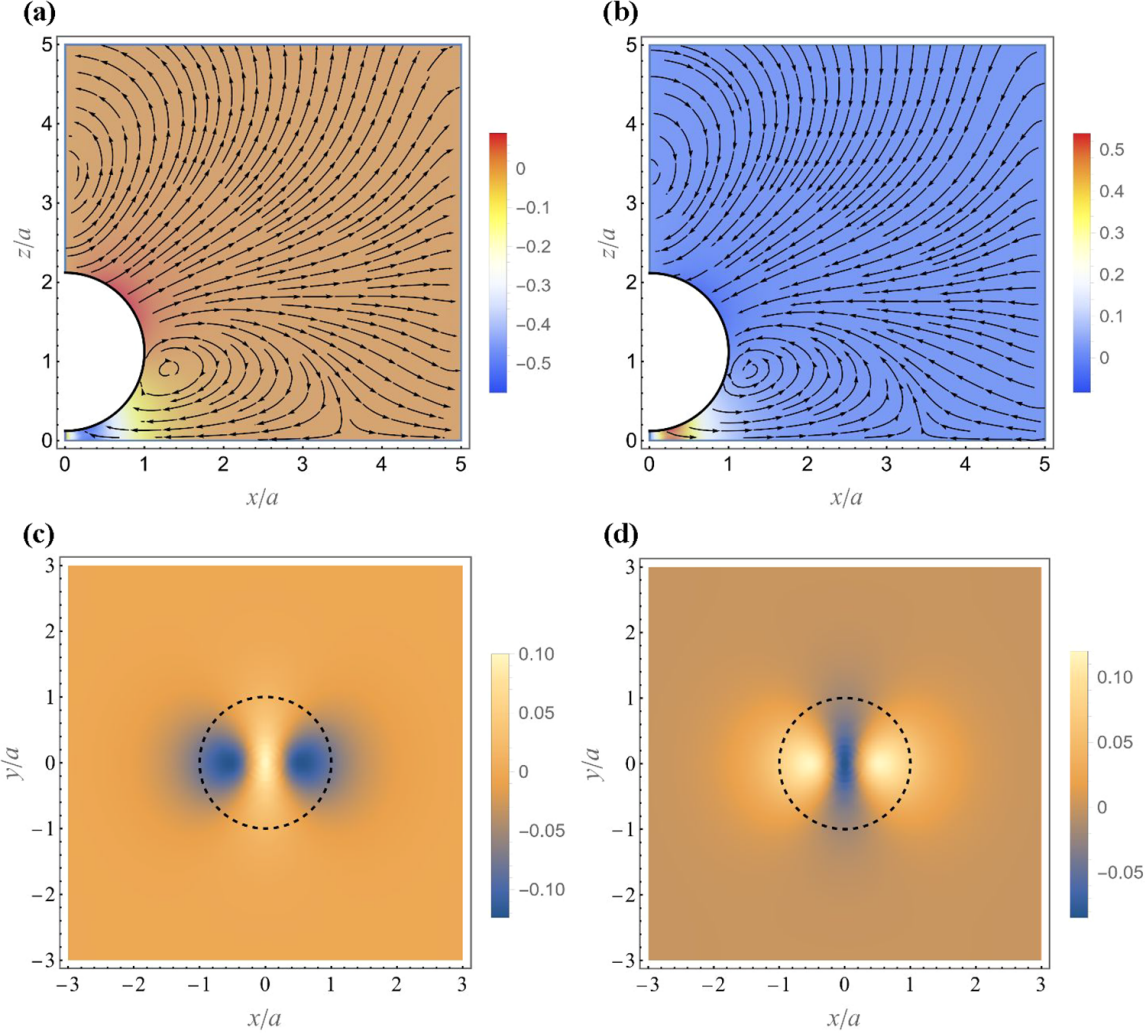
Upper panel shows the numerically computed perturbed flow field (streamlines) and pressure (color map, in units of *ην*_0_/*a* in the *xy*-plane) due to a freely suspended particle (free to rotate and translate), which corresponds to the nonlocalized adsorption regime, with *ρ*_*s*_/ρ = 1.35 for *a*/δ = 0.03 located above the resonator at a scaled separation distance ϵ = 0.12 for (a) ω*t* = 0; (b) *ωt*= π/2. The lower panel shows the perturbed tangential stress (color map) at the resonator at *z*= 0 for (c) *ωt*= 0; (d) *ωt*= π/2. The dashed circle in the lower panels depict the projection of the particle boundary onto *z* = 0.

Recall that for *a*/δ ≪ 1 the flow is accurately described by the steady Stokes equations and the undisturbed (i.e., without the particle) unidirectional time-periodic flow above the resonator, υ = υ_0_ *x̂e* ^–*z*/δ^ cos­(*z*/δ – *ωt*) at distances *z* ∼ *a* ≪ δ can be closely approximately by a simple shear flow with a shear rate ∂υ_*x*_/∂*z* = υ_0_/δ at *ωt* = 0 and −υ_0_/δ at *ωt* = π/2, implying the time symmetry for the pressure, flow and stress disturbances, as can be readily seen in Figure 1.

Notice that the stress perturbation shown in Figure 1 (lower panel) is limited to a small area underneath the particle (i.e., the lubrication gap), while for the adsorbed particle, the fluid-mediated stress (see Figure S1, lower panel) extends over the areas significantly exceeding the particle cross-section area. Therefore, it is expected that the linear particle adsorption regime, characterized by the additivity of the contributions of single adsorbates, is expected to hold to much higher values of surface coverage than for the rigidly adsorbed particles.

It was shown in a previous publication in ref 56 that the asymptotic expression for the impedance due to a sparse monolayer of particles suspended in a liquid in near proximity to the resonator (i.e., in the limit ϵ ≪ 1 ≪ δ/*a*) has the following form:

1 $$\frac{Z}{\eta añ}≃\left[\frac{7.582\left(1+\epsilon \right)+1.593}{0.6376+0.200\;\ln\epsilon^{-1}}\right]\pi \lambda^{2}-\left(40.159+\xi \right)\lambda^{2}$$

where *ñ* is the particle areal number density, the parameter ξ = *m*/(ρ*a* ^3^) quantifies particle inertia and λ^2^ = −*ia* ^2^ω/ν. Notice that since λ^2^ is purely imaginary, the leading contribution to the real part of *Z*/(*ηañ*) reduces to $O\left({\left|\lambda \right|}^{3}\right)$ , implying that the dissipation shift is expected to be smaller in comparison to the frequency shift by a factor ∝|λ|, as found for the adsorbed particle in ref 38. Determining the analogous to eq 1 closed-form expression for the real part of *Z*/(*ηañ*) would require a higher-order expansion. The first term in the numerator of the fraction in the square brackets contributed by the particle translation is about 5 times larger than the second term due to the particle rotation. Notice that in the limit of vanishing proximity, ϵ → 0, the expression in the square brackets vanishes (logarithmically slow, ∼1/ln ϵ^–1^) and the impedance tends to that due to an adsorbed particle,^38^ given by the second term in the right-hand side of eq 1, i.e., *Z*_*a*_/(*ηañ*) = −(40.159 + ξ)­λ^2^.

The comparison of the analytical prediction in eq 1 vs the numerical results for a fixed proximity to the resonator ϵ = 0.1 is depicted in Figure 2. The agreement is excellent without any adjustable parameters. At this distance, the impedance due to a sparse monolayer of suspended particles with “soft” lubricated contact is about 2.6 times smaller than for a layer of adsorbed particles with stiff contact (red line) and about 3.1 times larger than predicted by the Sauerbrey equation. The numerical solution is obtained using the scheme proposed by Fouxon et al.,^55^ which employs the Finite Element Method (FEM) implemented in *Mathematica* 13.0. The details of the calculations, along with representative results, are provided in the Supporting Information. The comparison between the numerical results and the analytical prediction of eq 1 is shown in Figure 2.

**Figure 2:**
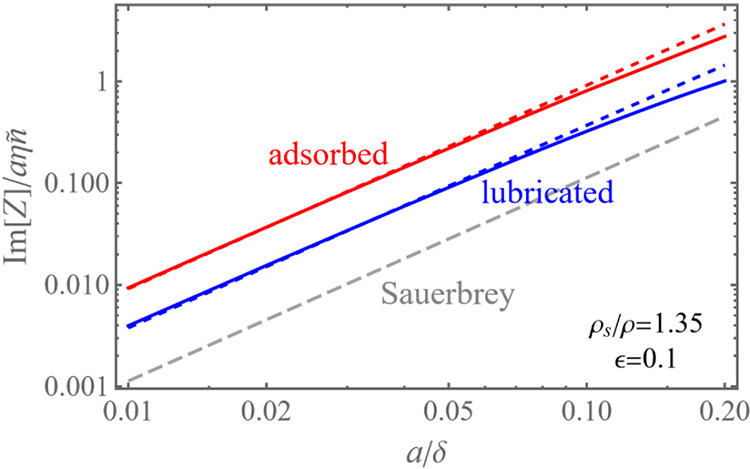
Numerically computed imaginary part of the dimensionless impedance, Im­[*Z*]/(*ηañ*) vs *a*/δ (log–log plot) due to a sparse layer of particles with density *ρ*_s_/ρ = 1.35, ϵ = 0.1; rigidly adsorbed particles (red solid curve) and freely suspended particles with lubricated contacts (blue solid curve) for the normalized separation distance. Short-dashed (blue and red) lines stand for the corresponding small-λ asymptotic approximations. The gray (long dashed) line shows the Sauerbrey impedance due to inertial mass of the particles in the layer.

The adsorption kinetics of protein molecules on the silica sensor was theoretically modeled using a hybrid approach where the bulk particle transport was described by the convective-diffusion equation with the nonlinear boundary condition derived from the random sequential model. A more extensive description of this approach is given in Supporting Information.

## 3 Results and Discussion

### 3.1 Bulk Particle and Substrate Characteristics

Apart from the density, which was 1.35 g cm^–3^, other physicochemical parameters of the protein molecules comprised the diffusion coefficient, directly measured by dynamic light scattering (DLS) for various pHs and NaCl concentrations of 10 and 150 mM, respectively. The hydrodynamic diameter, which is physically interpreted as the diameter of a sphere with the same diffusion coefficient as the protein molecule, was then calculated using the Stokes–Einstein relationship. To obtain information about the electrokinetic charge of the molecules, their electrophoretic mobility was measured using Laser Doppler Velocimetry (LDV) at different pH values. The zeta potential was calculated using the Henry formula.

Physicochemical parameters presented in Table 1 show that the hydrodynamic diameter ranged between 4.0, ca. 8.0 nm, and 21 nm for myoglobin, albumins, and fibrinogen molecules, respectively. In comparison, the equivalent sphere diameter *d*_*s*_, which corresponds to a sphere with the same mass as the protein molecule, was equal to 3.5, 5.4, and 9.3 nm for myoglobin, albumins, and fibrinogen, respectively. The advantage is that *d*_s_ is a uniquely defined parameter that only depends on the density and the protein molar mass.

**Table 1:** Physicochemical Characteristics of Protein Molecules: Myoglobin (MG), Bovine Serum Albumin (BSA), Human Serum Albumin (HSA) and Fibrinogen (FG); the Protein Density *ρ*_s_ = 1.35 g/cm^3^, at a Temperature of 298 K^a^

Protein	*M*_w_ (g mol^–1^)	*v*_*p*_ (nm^3^)	*D* (cm^2^ s^–1^)	*d*_H_ (nm)	*d*_s_ (nm)	*ζ* (mV)	Stability
MG	17,800	21.9	1.2 ± 0.05 × 10^–6^	4.2 ± 0.2	3.5	35 ± 3	pH < 5
						–19 ± 2	
BSA	66,400	81.7	6.1 ± 0.3 × 10^–7^	8.0 ± 0. 3	5.4	45 ± 3	pH < 5 and pH > 6
						–30 ± 2	
HSA	66,400	81.7	6.3 ± 0.3 × 10^–7^	7.8 ± 0.3	5.4	42 ± 3	pH < 5 and pH > 6
						–28 ± 2	
FG	340,000	418	2.3 ± 0.1 × 10^–7^	21 ± 0.1	9.3	45 ± 3	pH < 5 and pH > 6
						–30 ± 2	

a *M*_w_—molar mass of the protein, *v*_p_—molecule volume calculated as 10^21^ × *M*_w_/(ρ_s_ *N*_Av_) where *N*_Av_ is the Avogadro constant, *D*—the diffusion coefficient, *d*_H_—the hydrodynamic diameter, *d*_s_—the equivalent sphere diameter calculated as (6*ν*_p_/π)^1/3^, ζ—the zeta potential: upper and lower values correspond to pH 3.5 and 7.4, respectively, for 10 mM NaCl.

The stability of protein solutions was established through a series of additional DLS experiments, in which changes in the protein molecule hydrodynamic diameters at various pH levels were monitored over time. It was established that myoglobin solutions remained stable for the duration of a typical QCM run at pH below 5, while albumin and fibrinogen solutions were only unstable within the pH range of 5 to 6 (for NaCl concentrations of 10 and 150 mM). All molecules exhibited a positive zeta potential at pH 3.5 and a negative zeta potential at pH 7.4, regardless of the NaCl concentration (10 and 150 mM) or the presence of PBS buffer (Table 1). On the other hand, the zeta potential of silica plates determined by the streaming potential method was negative for pH values ranging from 3 to 10, with values of −15 and −50 mV at pH 3.5 and 7.4, respectively.^35^

### 3.2 QCM Measurements of Protein Adsorption/Desorption Kinetics

Several QCM runs were performed for each protein under various pHs, NaCl concentrations, bulk solution concentrations, and flow rates. The frequency Δ*f* and the dissipation Δ*D* shifts were registered for various overtones (denoted by *n*_0_ as a function of time. After the signals were established, desorption runs were initiated, in which a pure electrolyte solution of the same pH, ionic strength, and flow rate was flushed through the cell. The frequency shifts were expressed in the normalized form −Δ*f*/*n*_0_, and the dissipation changes were converted to the bandwidth changes, i.e., $-\Delta \Gamma =\frac{1}{2}f_{0}\Delta D$ , where *f*_0_ is the fundamental frequency of the quartz sensor equal to 5 × 10^6^ Hz.^56,57^ The results acquired for fibrinogen are shown in Figure 3 as the dependence of −Δ*f*/*n*_0_ on the time for the entire adsorption run (left-hand side part) and for the shorter time period (right-hand side). One should notice two characteristic features of these runs (i) the differences in the −Δ*f*/*n*_0_ signal among the overtones were insignificant for the entire range of the adsorption time, and (ii) the shifts of −Δ*f*/*n*_0_ were minor upon switching to the desorption mode, where the pure electrolyte was flushed through the cell.

**Figure 3:**
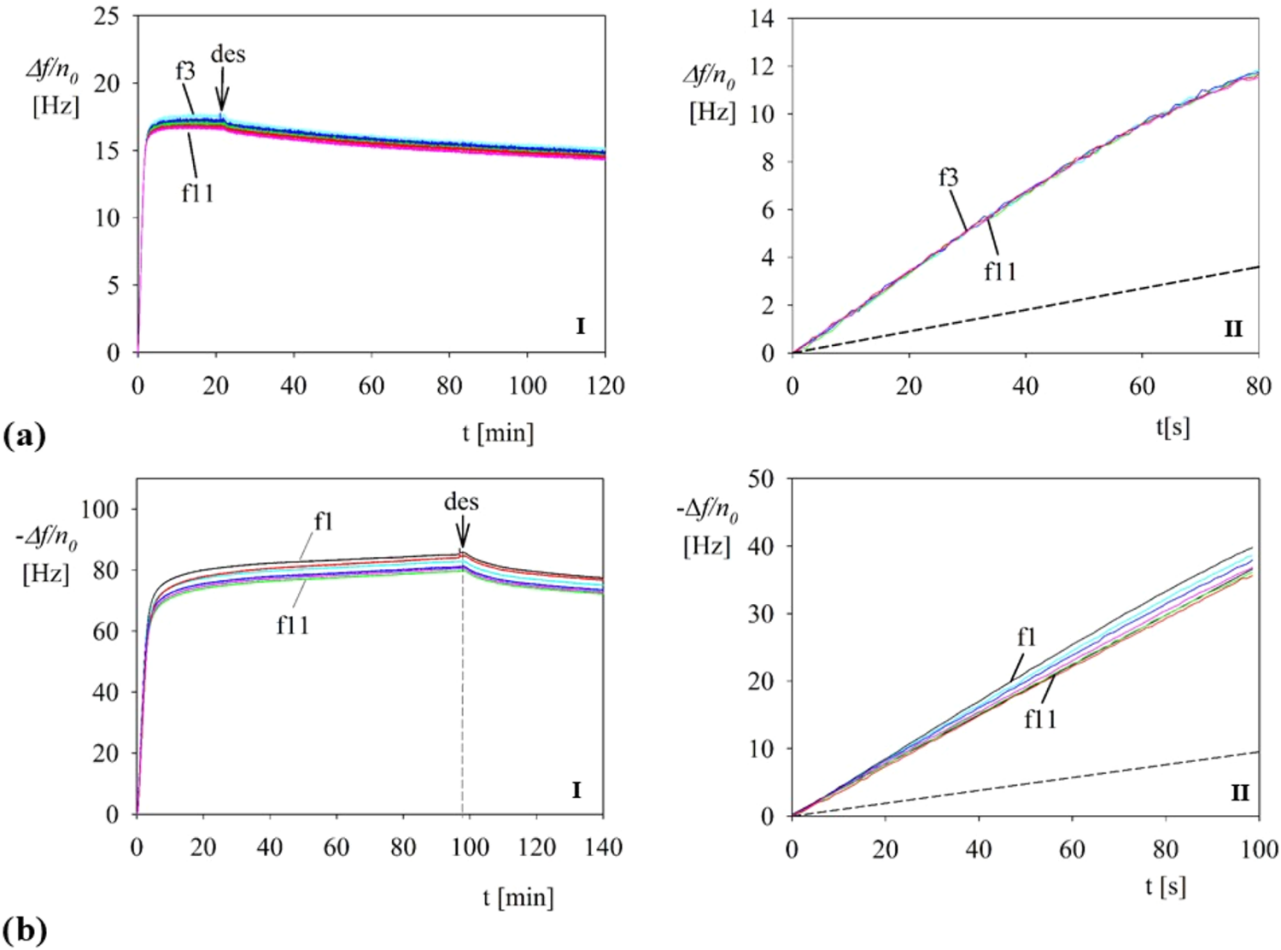
Primary results of QCM measurements acquired for fibrinogen shown as the dependence of the frequency shifts −Δ*f*/*n*_*o*_ (left panels (I)—long times, right panels (II)—short times) on the adsorption time; silica sensor: (a) *I* = 10 mM NaCl, pH 3.5, volumetric flow rate 2.5 × 10^–3^ cm^3^ s^–1^, bulk protein concentration 10 mg L^–1^; (b) 10 mM NaCl, pH 7.4, volumetric flow rate 2.5 × 10^–3^ cm^3^ s^–1^, bulk protein concentration 10 mg L^–1^. The dashed lines in Figures a (II) and b (II) represent the frequency shift −Δ*f*_s_(*t*) corresponding to the real coverage of the protein calculated from the RSA model.

It should also be observed (right panels of Figure 3) that at both pHs the −Δ*f*/*n*_0_ shifts initially increased in a linear way with time, and the difference in the slope between the first and the 11th overtone amounting o ca. 5% for longer times. It is also interesting o mention that the requency shift −Δ*f*_s_(*t*) corresponding to the real coverage of fibrinogen calculated from the RSA model (see the dashed lines in Figure 3a­(II) and b­(II)) was many times smaller than the QCM frequency shifts for this time range.

Analogous results acquired for myoglobin and albumin are shown in the Supporting Information.

As far as the ΔΓ signal is concerned, its changes with time were more than an order of magnitude smaller than the corresponding −Δ*f*/*n*_0_ shifts for all proteins (Supporting Information).

To quantitatively analyze the kinetic runs derived from QCM measurements it is useful to introduce the impedance concept, a quantity which characterizes the sensitivity of the sensor.^58,59^ Accordingly, the imaginary component of the impedance, pertinent to the frequency shifts Im­[*Z*(*t*)], can be calculated as a function of time using the following constitutive formula (Supporting Information)­

2 Im[Z(t)]=πZqnofo[−Δf(t)no]

where *Z*_q_ is the acoustic impedance of quartz equal to 8.8 × 10^6^ kg m^–2^ Hz^–1^, ref 21.

It is convenient to express the impedance using the normalized form using the inertia load *ωM*(*t*) = 2π*n*_0_ *f*_0_ *M*(*t*) as the scaling parameter, where *M*(*t*) is the real mass of a protein layer per unit area, often referred to as the dry mass.

In consequence, using eq 2, the normalized impedance becomes

3 $${\mathrm{Z̅}}_{\mathrm{im}}\left(t\right)=\frac{Z_{q}}{2f_{0}^{2}M\left(t\right)}\left[-\frac{\Delta f\left(t\right)}{n_{o}}\right]=\frac{M_{Q}\left(t\right)}{M\left(t\right)}$$

where

4 $$M_{Q}\left(t\right)=-\frac{C_{s}\Delta f\left(t\right)}{n_{o}}$$

is the apparent QCM mass coverage referred to as the ‘wet’ mass, and *C*_s_ = *Z*_q_/2*f*_0_^2^ is the Sauerbrey constant equal to 0.177 mg m^–2^ Hz^–1^ for *f*_0_ = 5 × 10^6^ Hz.

From eq 4 one can predict that the experimental impedance in the limit of short time where −Δ*f*(*t*)/*n*_*o*_ = *s*_lQ_(*n*_*o*_)*t*, and *M*(*t*) = *s*_l_ *t* can be determined from the formula

$${\mathrm{Z̅}}_{\mathrm{im}}^{0}={\left(\frac{dM_{Q}}{dt}\right)}_{t\to 0}{/(\frac{dM}{dt})}_{t\to 0}=C_{s}s_{\mathrm{lQ}}(n_{o})/s_{l}$$ 5

where *s*_lQ_(*n*_o_), *s*_l_ are the corresponding slopes of these linear dependencies. In this case, *Z̅*_*im*_^0^ is independent of the time. The *s*_l_ slope can be acquired either via simultaneous measurements of the adsorption kinetics by a complementary experimental method as was done in refs 20,21 or from the numerical solution of the convective mass transfer equation using the RSA approach as done in this work.

It should be mentioned that the relationship between the normalized impedances *Z̅*_*im*_^0^ and *Z*/(*ηañ*) appearing in eq 1 is the following

6 $${\mathrm{Z̅}}_{\mathrm{im}}^{0}=\frac{3}{8\pi }{\left(\frac{\delta }{a_{s}}\right)}^{2}\left(\frac{\rho }{\rho_{s}}\right)\frac{Z}{\eta a_{s}ñ}$$

where it is assumed *a* = *a*_s_ = *d*_s_/2.

Therefore, considering eq 6 and eq 1, the analytical expression for the *Z̅*_*im*_^0^ impedance in the low coverage limit can be expressed as

7 $${\mathrm{Z̅}}_{\mathrm{im}}^{0}=Z_{0}\left[1-\frac{3\rho_{s}F_{1}\left(\epsilon \right)}{4\rho Z_{0}}\right]$$

where $Z_{0}=1+9.6\frac{\rho }{\rho_{s}}$ , and

8 $$F_{1}\left(\epsilon \right)=\left[\frac{7.582\left(1+\epsilon \right)+1.593}{0.6376+0.200\ln\epsilon^{-1}}\right]$$

It is worth underlining that the theoretical impedance predicted from eq 7 for a fixed protein density depends only on the parameter ϵ = 2*h*/*d*_s_, where *h* is the minimal distance between the molecule and the sensor.

A comparison of experimental results obtained for the proteins (points) with the theoretical results derived from eq 7 (solid lines) is presented in Figure 5 as the dependence of *Z̅*_*im*_^0^ on *a*_s_/δ. The parameter ϵ was calculated assuming that *h* = 0.4 nm, which corresponds to the average roughness of the silica sensor in the length scale comparable with the protein molecule size (Supporting Information). For comparison, the hydrated ion diameters are 0.24 and 0.32 nm for Na^+^ and Cl^–^, respectively.^60^

As shown in Figure 4, the agreement between theoretical predictions derived under the assumption of a nonlocalized adsorption regime (solid green lines) and experimental results is satisfactory, except for fibrinogen at pH 7.4, where the theoretical results underestimate the experimental ones. This effect can likely be attributed to the prevailing end-on orientation of adsorbed fibrinogen molecules, as demonstrated in previous work.^35^ It is also worth observing that in all cases the experimental impedances were independent of *a*_s_/δ, i.e., on the overtone number. It should also be emphasized that the experimental impedances were considerably smaller than those predicted, assuming a rigid contact of molecules with the sensor (represented by the dashed red line in Figure 3) described by the equation

$${\mathrm{Z̅}}_{\mathrm{im}}^{0}=Z_{0}-C_{a}a_{s}/\delta$$ 9

where *C*_a_ = 12.

**Figure 4:**
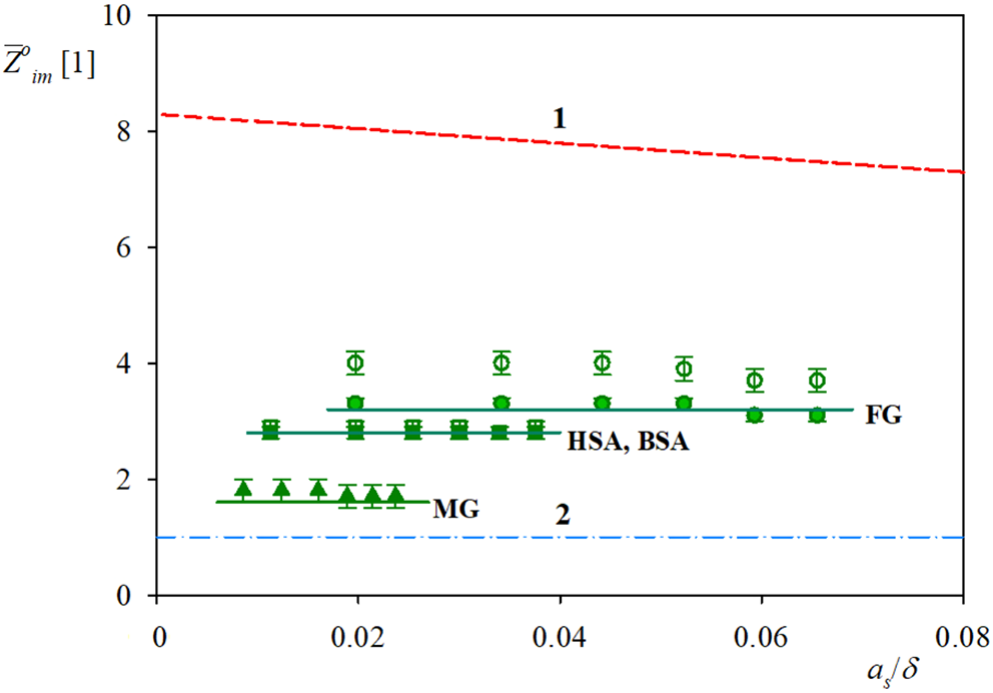
Dependence of normalized impedance *Z̅*_*im*_^0^ on the normalized particle size. *a*_s_/δ. The points show experimental results obtained for various proteins (overtones 1–11): Open circle: fibrinogen (FG) pH 7.4, Green circle solid: FG pH 3.5, ϵ = 0.085; Open square: albumin (HSA), Green square solid: albumin (BSA), ϵ = 0.14 Green triangle solid: myoglobin (MG), ϵ = 0.24. The solid green lines show the theoretical results derived from eq 7. The dashed red line 1 shows the results predicted for the rigid contact of the protein molecule with the sensor, and the blue dashed/dotted line 2 shows the results pertinent to the real coverage of the proteins, i.e., to the pure inertia load.

Therefore, the results shown in Figure 4 furnish evidence supporting the nonlocalized adsorption mechanism of the protein molecules on the silica sensor. This conclusion agrees with the results obtained in ref 61, where molecular dynamics modeling was applied to investigate BSA molecule adsorption at a silica/NaCl electrolyte interface. It was demonstrated *inter alia* that whereas the perpendicular component binding energy was adequate to ensure an irreversible adsorption, the lateral component was significantly smaller, enabling surface diffusion of the molecules. It was also shown that the minimum approach distance of the BSA molecule in the F-form was ca. 0.4 nm. However, this conclusion remains strictly valid for sensors of low roughness whose correlation length is significantly larger than the protein characteristic dimension and which are characterized by perfectly homogeneous surface properties, particularly the charge distribution.

To facilitate the comparison of results acquired in this work with literature data, they are alternatively presented in Figure 5 as the dependence of the *Z̅*_*im*_^0^ impedance on the equivalent sphere size *d*_s_ = 2*a*_s_. The advantage of this parameter is that it only depends, for a fixed density, on the molar mass of the protein. For the density of 1.35 g cm^–3^ one can uniquely calculate *d*_s_ (in nm) from the formula

10 $$d_{s}=2a_{s}=0.133M_{w}^{1/3}$$

where the molar mass is expressed in g mol^–1^.

**Figure 5:**
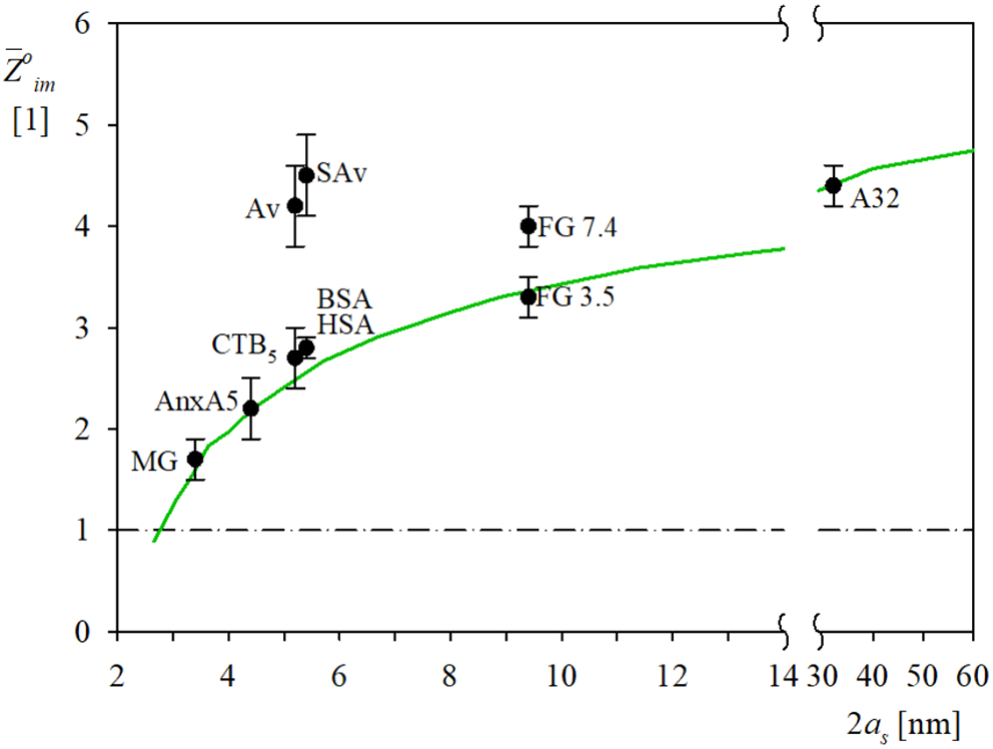
Dependence of normalized *Z̅*_*im*_^0^ impedance on the equivalent sphere diameter of protein molecules 2*a*_s_, for the minimum distance *h* = 0.4 nm. The points represent experimental results obtained for various proteins: fibrinogen, albumins, myoglobin (this work), annexin (AnxA5), the cholera toxin subunit (CTB_5_),^21^ avidin (Av) and streptavidin (SAv).^20^ The reference result for the spherical amidine polymer particles (A32) is also shown. The solid green line represents the theoretical results derived from eq 7 and the dashed-dotted line shows the results pertinent to the pure inertia load.

One should mention that *d*_s_ is a good measure of the true shape of globular (compact) protein molecules such as myoglobin and albumins, where it approaches the corresponding hydrodynamic diameter values. However, for elongated molecules such as fibrinogen, the *d*_s_ value is close to the shorter axis of the molecule, equal to ca. 7 nm.^35^

One can observe in Figure 5 that the theoretical results derived from eq 7 assuming the nonlocalized adsorption regime (lubricated contact) adequately reflect experimental data for most proteins comprising annexin (AnxA5) and the cholera toxin subunit (CTB_5_) investigated using simultaneous QCM and ellipsometric measurements.^21^ A more significant deviation, exceeding the experimental error, is only observed for avidin (Av) and streptavidin (SAv), where simultaneous QCM and reflectometric measurements were applied.^20^ This was probably caused by stronger contact with the lipid substrate of larger protein oligomers. It is also worth mentioning that the results obtained for amidine polymer particles (A32) of a spherical shape and the size of 32 nm quantitatively agree with the theory.

On the other hand, analogous results showing the dependence of *Z̅*_*im*_^0^ on the minimum approach distance calculated using the hydrodynamic diameter of the protein as a scaling parameter, where ϵ = 2*h*/*d*_H_ are presented in the Supporting Information.

However, one should mention that the experimental impedance shown in Figure 5 corresponds to the low coverage range, where the QCM frequency shifts, $-\frac{\Delta f\left(t\right)}{n_{o}}$ and Δ*f*_s_(*t*), corresponding to the pure inertia load remain linear functions of time. For higher surface coverage, the impedance is expected to decrease due to the perturbation of the oscillatory flow near the sensor by adsorbed molecules. To determine the significance of this effect the experimental frequency shifts acquired for arbitrary times were analyzed using the method previously applied in refs 38,56 for polymer and noble metal nanoparticles. Accordingly, for a given coverage, the adsorption time *t*_m_ is calculated by a numerical inversion of the *M*(*t*) function known from the RSA modeling. Then, the impedance is calculated from the constitutive dependence

11 $${\mathrm{Z̅}}_{\mathrm{im}}\left(M\right)=M_{Q}\left(t_{m}\right)/M\left(t_{m}\right)$$

where *M*_Q_(*t*_m_) = −*C*_s_Δ*f*(*t*_m_)/*n*_o_

Dependences of the normalized impedance *Z̅*_*im*_ on the real (dry) protein coverage acquired in this way are shown in Figure 6. As can be seen, in all cases the decrease in the impedance with the coverage was a rather insignificant amounting to ca. 15% at *M* = 1 mg m^–2^ for albumins and fibrinogen at pH 3.5. The experimental results were adequately fitted by linear dependencies, i.e.,

12 $${\mathrm{Z̅}}_{\mathrm{im}}={\mathrm{Z̅}}_{\mathrm{im}}^{0}\left(1-C_{1}M\right)$$

where the *C*_1_ constant is expressed in m^2^ mg ^–2^. Values of *Z̅*_*im*_^0^ and *C*_1_ for various proteins are given in Table 2.

**Figure 6:**
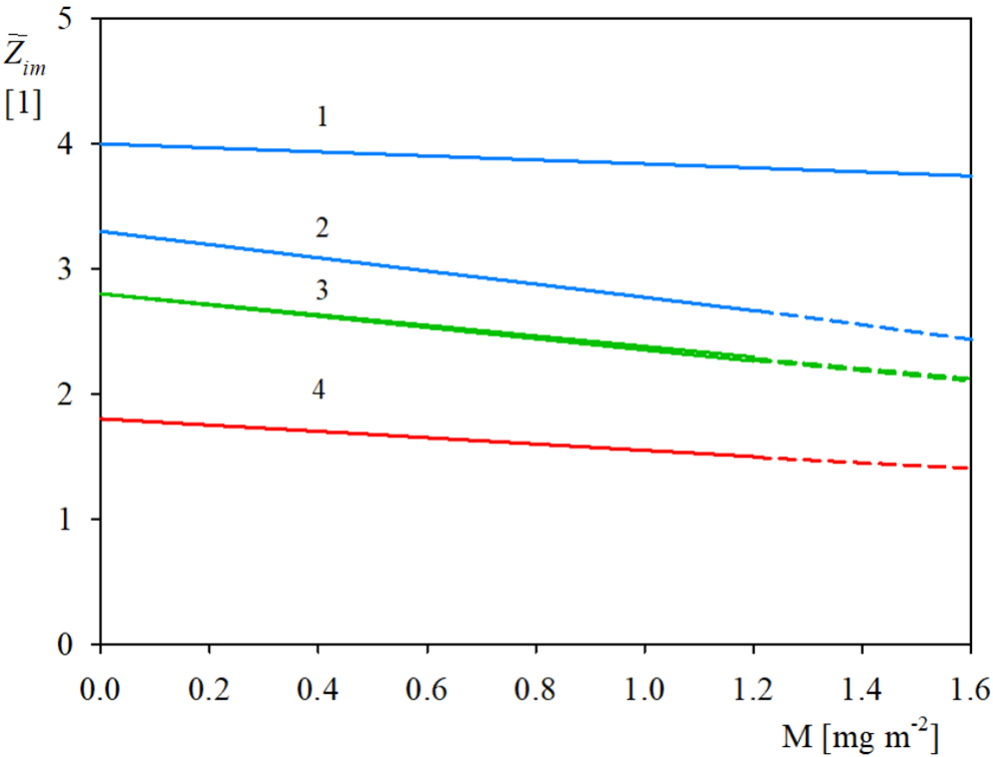
Dependence of the experimental impedance *Z̅*_*im*_ on the mass coverage of protein molecules: 1. Fibrinogen pH 7.4, 2. Fibrinogen pH 3.5, 3. Albumins (HSA, BSA); 4. Myoglobin. The solid lines represent linear fits of the experimental results calculated from eq 12.

**Table 2:** Basic Parameters for Calculating the Real Adsorption Kinetics of Protein Molecules Using QCM Measurements (For the 3rd Overtone), Silica Sensor^a^

Protein	*∈* (1)	*Z̅*_*im*_^0^ (1)	*C*_1_ (m^2^ mg^–2^)	*C*_s_′ (mg m^–2^ Hz^–1^)	Remarks
MG	0.24	1.8 ± 0.3	0.14	0.0983	pH 3.5
BSA	0.14	2.8 ± 0.2	0.15	0.0632	pH 3.5
HSA	0.14	2.8 ± 0.2	0.16	0.0632	pH 3.5
FG	0.085	3.3 ± 0.2	0.16	0.0536	pH 3.5
FG	0.085	4.0 ± 0.2	0.4	0.0442	pH 7.4

a ϵ = *h*/*a*_s_—scaled minimum distance of molecule from the sensor, *Z̅_im_* ^0^—scaled impedance for low coverage, *C*_1_—the constant in the impedance correction function: *Z̅*_*im*_(*M*) = *Z̅*_*im*_^0^ (1 – *C*_1_ *M*), *M*—protein dry coverage, *C*_s_′ = *C*_s_/*Z̅*_*im*_^0^—modified Sauerbrey constant for the fundamental frequency *f*_0_ = 5 × 10^6^ Hz.

Considering that M = *M*_Q_/*Z̅*_*im*_^0^ and using eq 12, one obtains the following expression enabling direct calculations of the real coverage:

$$M=\frac{1-{\left(1-\frac{4C_{1}M_{Q}}{{\overset{¯}{Z}}_{\mathrm{im}}^{0}}\right)}^{1/2}}{2C_{1}}$$ 13

Note that eq 13 is valid if 4*C*_1_ *M*_Q_/*Z̅*_*im*_^0^ < 1.

If 4*C*_1_ *M*_Q_/*Z̅*_*im*_^0^ is much smaller than unity, eq 13 simplifies to the following form

14 $$M\left(t\right)=\frac{M_{Q}\left(t\right)}{{\mathrm{Z̅}}_{\mathrm{im}}^{0}}\left[1+C_{1}\frac{M_{Q}\left(t\right)}{{\mathrm{Z̅}}_{\mathrm{im}}^{0}}\right]$$

where *M*_Q_(*t*)/*Z̅*_*im*_^0^ = *C*_s_^′^[−Δ*f*(*t*)/*n*_o_], and *C*_s_^′^ = *C*_s_/*Z̅*_*im*_^0^ is the modified Sauerbrey constant whose values are also given in Table 2 for *n*_o_ = 3. As can be noticed, for the protein molar mass within the range of 50,000 to 300,000 g mol^–1^ (where or *d*_s_ varies between 5 and 9 nm) the modified Sauerbrey constant amounts to approximately one-third of the common Sauerbrey constant equal to 0.177 mg m^–2^ Hz^–1^ (for *f*_0_ = 5 × 10^6^ Hz).

It is worth underlining that because of a simple form, eq 14 allows reliable calculation of the real adsorption kinetics of protein molecule using the frequency change −Δ*f*(*t*)/*n*_o_ for arbitrary overtone (because *Z̅*_*im*_^0^ was shown to be independent of the overtone), transforming QCM into a quantitative technique.

To confirm this fact, the kinetic QCM results obtained for BSA for various bulk concentrations and converted to the real coverage vs the time dependencies using eq 14 are shown in Figure 7. The results illustrate two important points: (i) there was no aggregation of albumin molecules because the *M* vs time *t* dependences remained linear for initial times matching the maximum adsorption rate pertinent to the QCM cell, and (ii) the maximum coverage was independent of the bulk protein concentration and was equal to 1.2 mg m^–2^ matching the maximum coverage obtained from reflectometry measurements. These facts confirm that BSA adsorption was monolayer characterized by a high-affinity isotherm.

**Figure 7:**
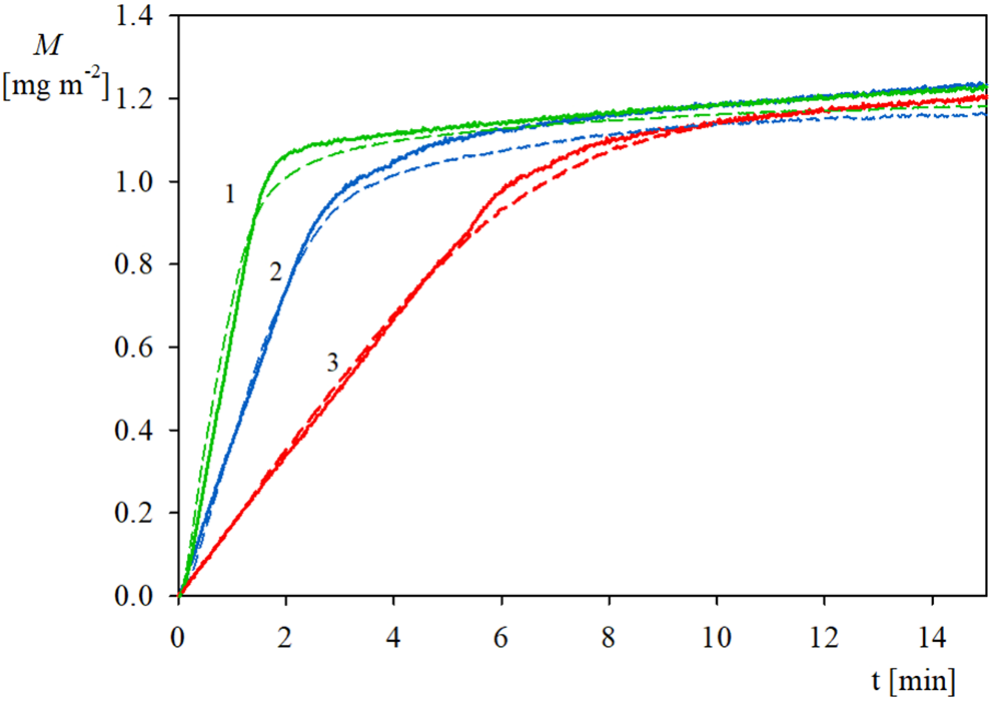
Kinetics of the BSA molecule adsorption for various bulk concentrations expressed as the dependence of the mass coverage *M* on the adsorption time *t*. The solid lines denote the results calculated from eq 14 for the third overtone; silica sensor, pH 3.5, 150 mM NaCl, flow rate 1.25 × 10^–3^ cm^3^ s^–1^. (1) Bulk concentration 10 mg L^–1^. (2) Bulk concentration 5 mg L^–1^. (3) Bulk concentration 2 mg L^–1^. The dashed lines represent the adsorption kinetics experimentally derived from reflectometry measurements for the silica sensor, conducted at the same mass transfer rates as in the QCM measurements.

Other QCM kinetic runs acquired for HSA under various NaCl concentrations and transformed to the real protein coverage using eq 14 are shown in Figure 8. As can be observed, the *M* vs *t* dependences remained linear for initial times, independently of the ionic strength, which indicates that the adsorption rate was controlled by the bulk transport in accordance with the RSA model. In contrast, the maximum protein coverage abruptly increased with ionic strength, attaining 0.25, 0.6, and 1.2 mg m^–2^ for 1, 10, and 150 mM NaCl concentrations. These results agree with theoretical results calculated using the effective hard particle approach^62^ considering the lateral electrostatic interactions among adsorbed molecules. The range of these repulsive interactions is comparable with the Debye screening length, which was equal to 31 and 3.1 and 1 nm for 0.1 nm mM and 10 mM NaCl, respectively. It should also be noted that these maximum coverages agree, within experimental error bounds, with those obtained by OWLS.

**Figure 8:**
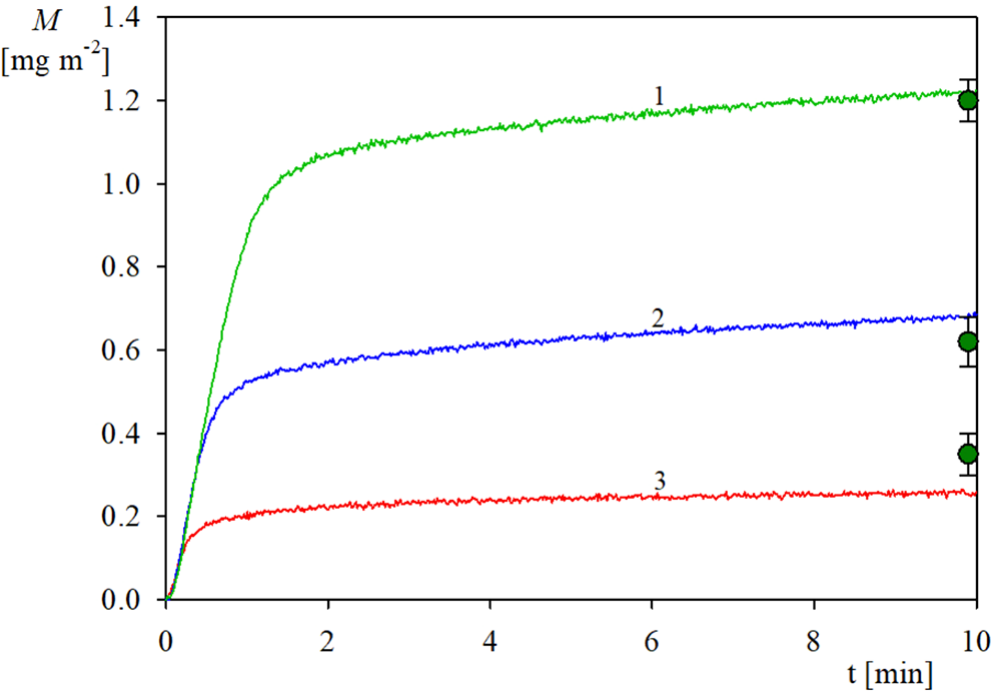
Kinetics of the HSA molecule adsorption at various NaCl concentrations (ionic strength) expressed as the dependence of the particle mass coverage *M* on the adsorption time *t*. The solid lines denote the results calculated using eq 14 for the third overtone; silica sensor, pH 3.5, bulk concentration 10 mg L^–1^, 150 mM NaCl, flow rate 1.25 × 10^–3^ cm^3^ s^–1.^, the arrow shows the beginning of the desorption run. Meaning of the curves: (1) 150 mM NaCl, (2) 10 mM NaCl, (3) 1 mM NaCl. The points show the maximum layer coverage experimentally derived from OWLS and theoretically calculated using the effective hard sphere approach.

One could argue that the results shown in Figures 7–8 confirm that the QCM measurements, properly interpreted using the impedances calculated from eq 14, can provide valid information about protein molecule adsorption at silica/electrolyte interfaces.

## 4 Conclusions

The mechanism of protein adsorption on silica/electrolyte surfaces was determined by experimental investigations and theoretical modeling. In particular, QCM measurements enabled the determination of the silica sensor impedance, which was weakly dependent on the overtone number and monotonically increased with the protein size expressed in terms of the equivalent sphere diameter. These experimental results were interpreted in terms of the random sequential adsorption modeling and the hydrodynamic theory assuming a lubricated contact of molecules with the substrate. A useful analytical expression in eq 7 enabled calculation of the impedance for a broad range of protein sizes. The accuracy of this formula was confirmed by numerical calculations performed by the finite element method.

It was shown experimentally that the impedance slightly decreased with the protein coverage, which was accounted for introducing a linear correction function, given by eq 14. This closed-form expression enabled a quantitative conversion of the QCM frequency signals to the real coverage of protein layers. In consequence, the adsorption/desorption kinetics of proteins acquired by QCM can yield reliable information about the protein adsorption mechanism. It is worth emphasizing that our approach represents a significant improvement for the entire range of protein coverage (up to the monolayer limit) compared to the Sauerbrey model commonly used in the literature, which introduces the systemic error ranging from ca. 80 to 300% for myoglobin and fibrinogen, respectively.

Using this approach, a nonlocalized adsorption mechanism of investigated proteins on the silica/electrolyte surface was confirmed perhaps with the exception of fibrinogen at pH 7.4, where the hydrodynamic effect can play a more significant role because of the prevailing end-on orientation of molecules. It was also shown that the adsorption of albumin molecules was of a monolayer type with the maximum coverage independent of the bulk protein concentration. However, the coverage increased with electrolyte concentration unvealing a significant role of lateral electrostatic interactions of protein molecules within adsorbed layers.

## Supplementary Material



## Notes

Numerical calculations topographic characteristics of sensors modeling particle deposition kinetics—the hybrid RSA approach interpretation of the quartz microbalance results (PDF)
