# Supplementary material for: Protein Adsorption Kinetics on Silica: Theoretical Modeling and Experiments
Source: Langmuir. 2026 Apr 6;42(16):11146–56. doi: 10.1021/acs.langmuir.6c00098 (PMC13298892; doi:10.1021/acs.langmuir.6c00098)
Supplement: Supplementary file 1 [file la6c00098_si_001.pdf]

SUPPORTING INFORMATION

Protein Adsorption Kinetics on Silica: Theoretical Modeling and Experiments

Monika Wasilewska [1]*, Agata Pomorska Gawel[1], Maria Morga[1],
Małgorzata Nattich-Rak[1], Zbigniew Adamczyk[1]*
Boris Y. Rubinstein[2], Alexander M. Leshansky[3]

[1] Jerzy Haber Institute of Catalysis and Surface Chemistry, Polish Academy of Sciences, Niezapominajek 8, 30 - 239 Krakow, Poland;

[2] Stewers Institute for Medical Research, Kansas City, Missouri 64110, United States;

[3] Department of Chemical Engineering, Technion – IIT, Haifa 32000, Israel

*Corresponding authors, e-mails:

Monika Wasilewska, email: monika.wasilewska@ikifp.edu.pl

Zbigniew Adamczyk, email: zbigniew.adamczyk@ikifp.edu.pl

Table of contents:

1. Numerical Calculations
2. Topographic Characteristics of Sensors
3. Modeling Protein Adsorption Kinetics-the Hybrid RSA Approach
4. Interpretation of the Quartz Microbalance Results

1. **Numerical Calculations**

Numerical calculations were carried out following the scheme proposed by Fouxon et al.[1] using the Finite Element Method (FEM) implemented in *Mathematica* 13.0. A fine mesh size was selected with a maximum cell size of $0.025a$ within the domain and $0.0125a$ (with a being the particle radius) along the boundaries to accurately resolve the flow in the lubrication gap separating the particle from the resonator. The FEM numerical results corresponding to the rigidly adsorbed particle are available in Leshansky et al.[2]

The numerically computed perturbed flow field (streamlines) and pressure due to a rigidly adsorbed particle (pertinent to the localized adsorption mechanism) for $a/\delta = 0.03$ and $\epsilon = 0.12$ are presented in Figure S1. Analogous results obtained for a freely suspended particle (pertinent to the non-localized adsorption mechanism) are shown in Figure 1, in the main text.

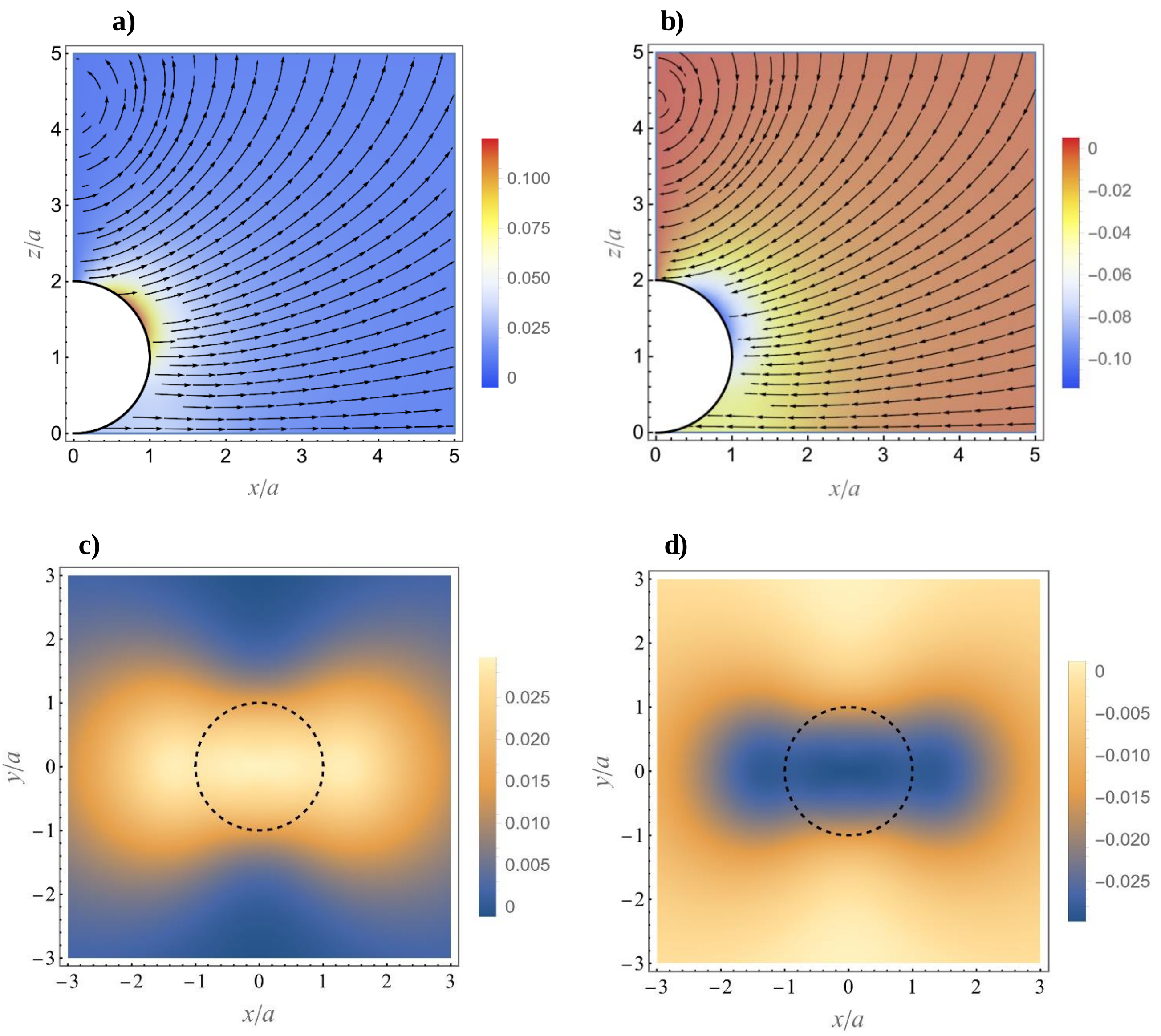

Figure S1: The upper panel shows the numerically computed perturbed flow field (streamlines) and (pressure map, in units of $\eta v_0/a$) in the xy-plane due to a rigidly adsorbed particle (for $\epsilon \approx 0$) with $\rho_s/\rho = 1.35$ for $a/\delta = 0.03$ (corresponding to HSA at 3rd overtone) located above the resonator at a scaled separation distance $\epsilon = 0.12$ for (a) $\omega t = 0$; (b) $\omega t = \pi/2$. The lower panel shows the perturbed tangential stress (colour map, in units of $\eta v_0/a$) at the resonator at $z = 0$ for (c) $\omega t = 0$; (d) $\omega t = \pi/2$. The dashed circle in the lower panels depict the projection of the particle boundary onto $z = 0$.

Notice that the magnitude of the tangential stress due to a rigidly adsorbed particle in Figures S1 (lower panel) is smaller than due to a freely suspended particle with lubricated contact (Figure 1, main text lower panel) while the resulting frequency shift in the former case is larger. This is so because in the case of the rigidly adsorbed particle, the major contribution to the signal comes from the contact force (i.e., the force the flow exerts on the particle, which is transmitted to the resonator via contact), while the fluid-mediated stress plays a minor role[1,2]. For the suspended particle (with no physical contact), the signal is solely due to the fluid-mediated stress perturbation. Notice also that the flow disturbance due to an adsorbed particle has a much longer range than that due to a freely suspended particle.

The dependence of the numerically computed imaginary part of the scaled impedance, $\mathrm{Im}[Z]\ /(\eta a\tilde{)}$, on the scaled radius a/δ is shown in Figure S2a for the rigid contact (red solid curve) and the lubricated contact (blue solid curve). The scaled minimal separation distance was equal to $\epsilon = 0.05$. One can observe that the numerical results agree with those calculated using the small-λ asymptotic approximations (dashed lines), which correspond to eq 1 (manuscript) for the lubricated contact. For a/δ smaller than 0.1, the agreement is quantitative. It is also worth mentioning that the impedances predicted in the case of the lubricated contact exceed several times the Sauerbrey impedance due to the particle inertia alone.

In Figure S2b, the numerically computed imaginary and real parts of the impedance are plotted vs. the scaled radius a/δ for $\epsilon = 0.05$ and the lubricated contact. Except for an adequate agreement of the numerical and analytical result (depicted by dashed lines), it is worth underlining that the real part of the impedance is smaller (in absolute terms) by a factor $\propto a/\delta$ in comparison to the imaginary part. Considering that the real and imaginary parts of the impedance correspond to the dissipation and frequency shifts, respectively, these calculations unequivocally confirm the advantage of the latter signal in electro-acoustic measurements because they ensure a higher sensitivity.

It is worth mentioning that numerical calculations performed for other minimum separation distances showed analogous trends, particularly an adequate agreement of the imaginary impedance with eq 1 (manuscript) especially for a/δ smaller than 0.1.

a)

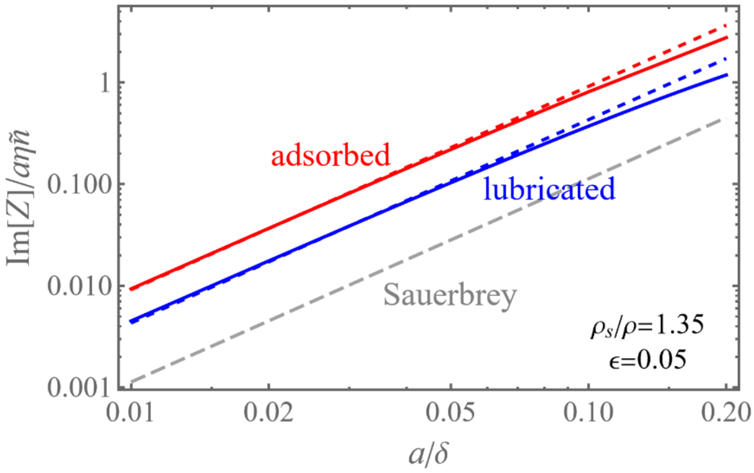

b)

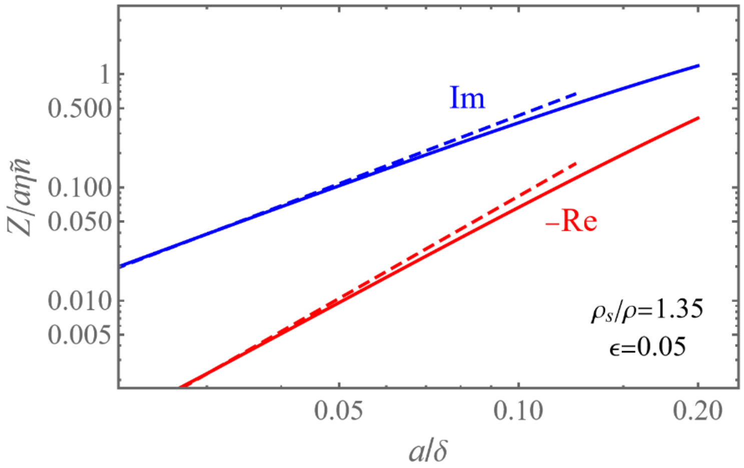

Figure S2. (a) Numerically computed imaginary part of the scaled impedance, $\mathrm{Im}[Z]/(\eta a\tilde{n})$ vs. a/δ (log-log plot) due to a sparse layer of particles with the scaled density $\rho_s/\rho = 1.35$; rigidly adsorbed particles (red solid curve) and particles with lubricated contacts (blue solid curve); the normalized minimal separation distance $\epsilon = 0.05$. The short-dashed lines stand for the corresponding small-λ asymptotic approximations calculated from eq 1 in the case of the lubricated contact. The grey (long-dashed) line shows the Sauerbrey impedance due to particles' solid inertia. (b) Real and imaginary parts of the impedance $Z/(\eta a\tilde{n})$ vs. a/δ (log-log plot) due to a sparse layer of particles with the scaled density $\rho_s/\rho = 1.35$ with lubricated contact for the normalized minimal separation $\epsilon = 0.05$; solid curves show the numerical results, dashed lines are the corresponding small-λ approximations (calculated from eq 1 for $\mathrm{Im}[Z]/(\eta a\tilde{n})$).

2. **Topographic Characteristics of Sensors**

The topography of the gold/silica sensor used in QCM measurements was determined by atomic force microscopy (AFM) imaging carried out under a semi–contact mode using the NT-MDT Solver BIO device with the SMENA SFC050L scanning head. A typical image of the gold/silica sensor with surface height profiles are shown in Figures S3a,b,c.

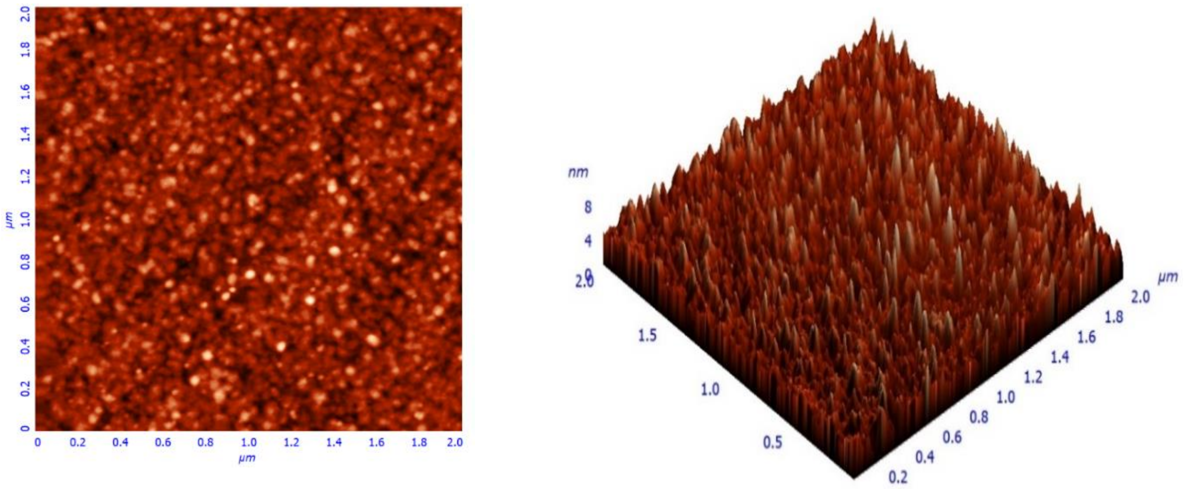

Figure S3a. AFM image of the gold/silica QCM sensor, the top (lhs) and the perspective (rhs) views.

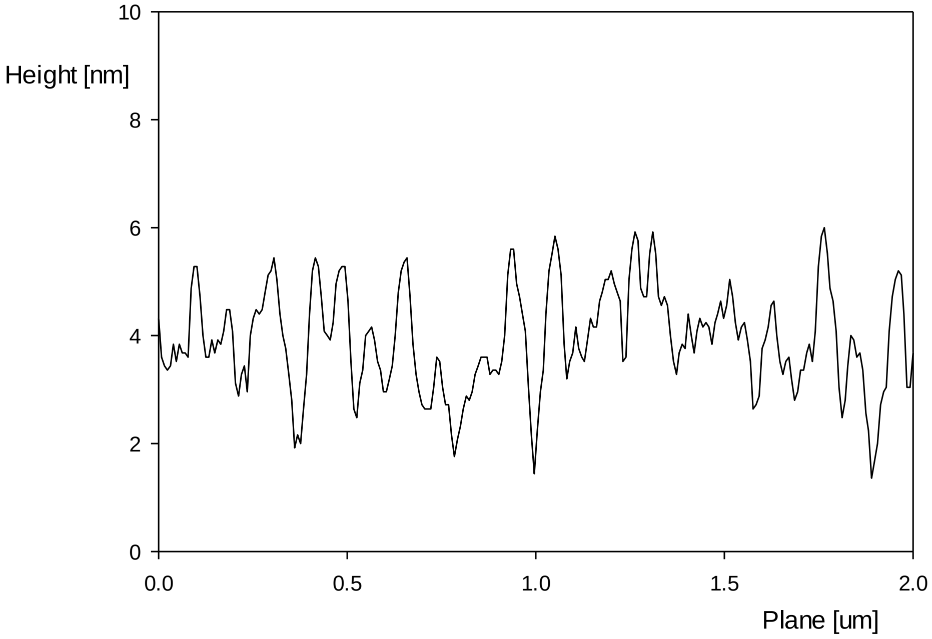

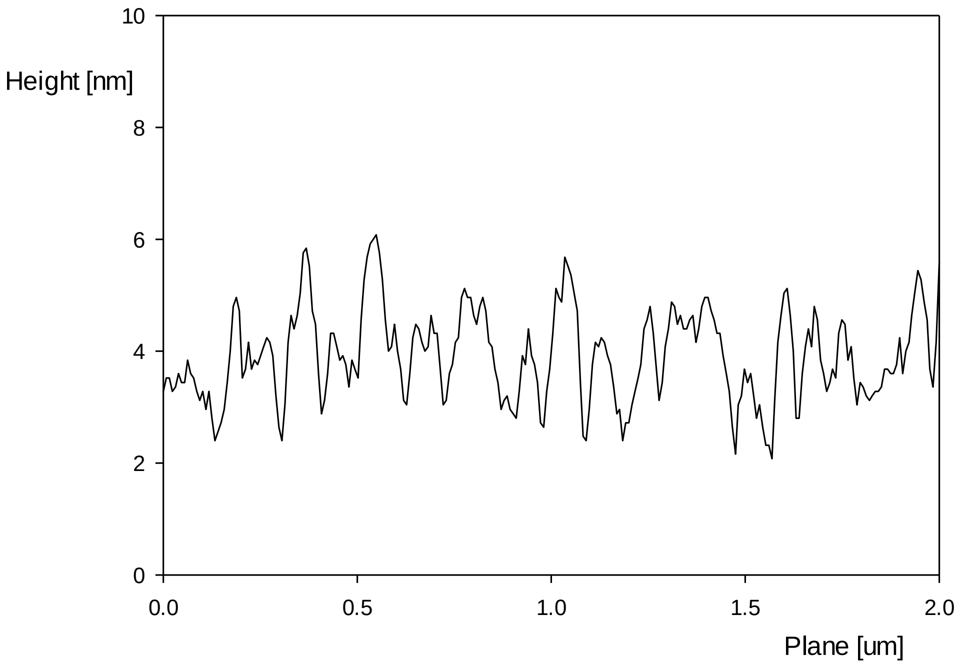

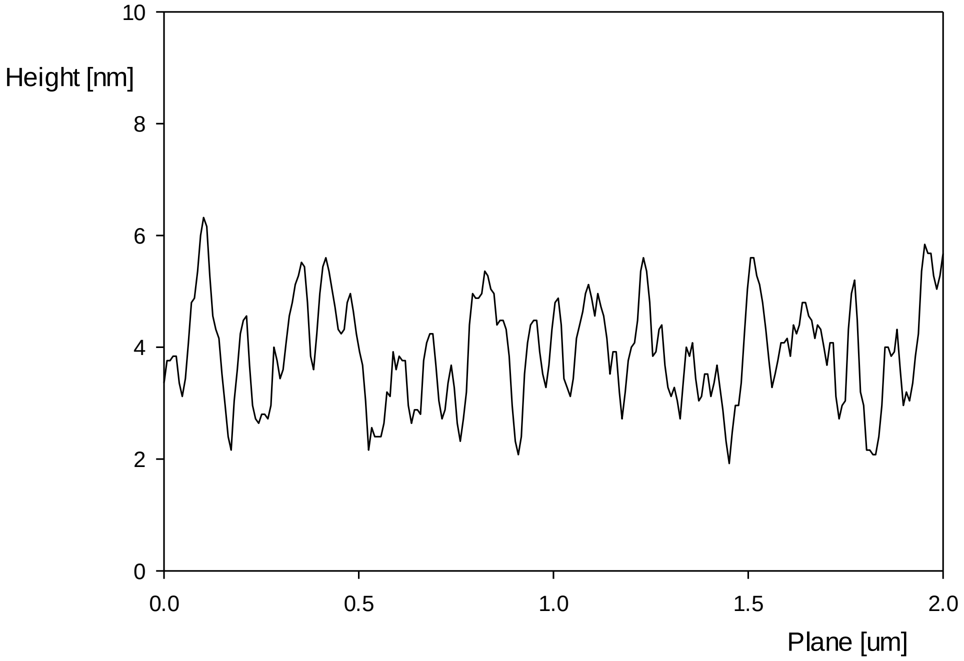

Figure S3b. AFM image of the gold/silica QCM sensor, examples of local surface height profiles.

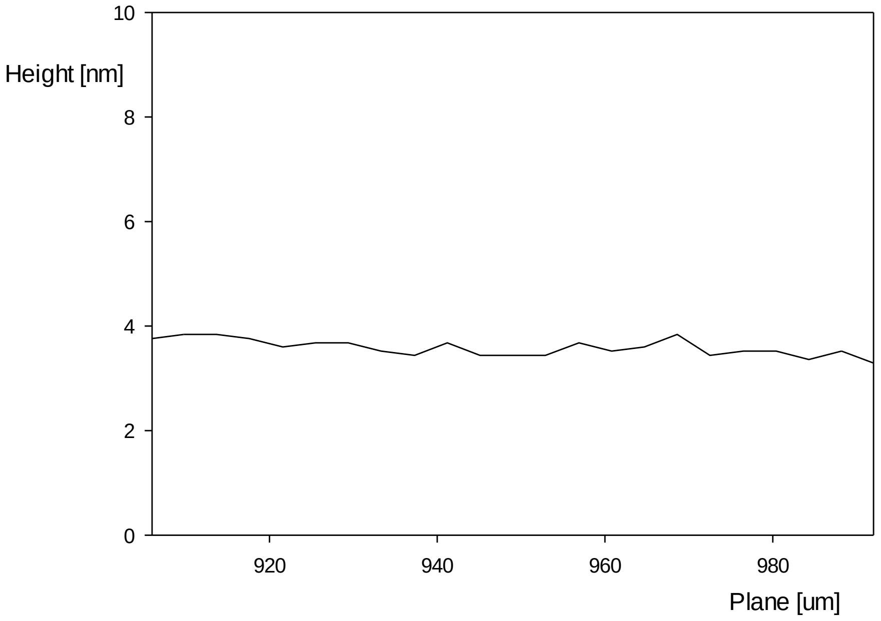

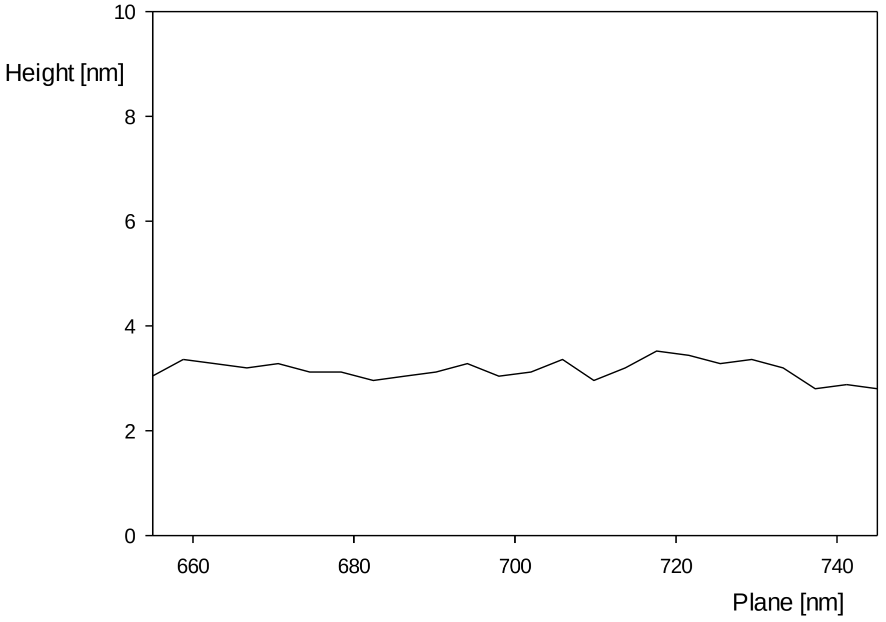

Figure S3c. AFM image of the gold/silica QCM sensor, examples of local height profiles in a large magnification of the horizontal axis.

Using the discrete set of surface heights h_i obtained from the AFM scanning, the average height ($\bar{h}$), the root mean square (rms) and the skewness (sk) of the sensor surface were calculated as follows[3]

$$\bar{h} = \frac{1}{N_i}\sum_{i=1}^{N_i}(h_i - h_0)$$
$$rms^2 = \frac{1}{N_i}\sum_{i=1}^{N_i}\left(h_1 - \bar{h}\right)^2 \qquad (S1)$$
$$sk = \frac{1}{rms^3 N_i}\sum_{i=1}^{N_i}\left(h_i - \bar{h}\right)^3$$

where h_0 is the reference height, and N_i is the number of mesh points of the AFM scan.

The roughness correlation length and its wavelength, which physically correspond to the average distance between two neighboring peaks of the surface height profiles, were also determined. These topographical parameters of the sensor are collected in Table S1. It is interesting to observe that the roughness wavelength is more than two orders of magnitude larger than the *rms* parameter, which means that the local surface roughness within the area between adjacent peaks is significantly smaller than 0.9 nm. To confirm this, a thorough topographical analysis of a fragment of sensor surfaces between the wavelength peaks was carried out; the corresponding AFM surface profiles are shown in Figure 2c. It was determined that the average surface height in these regions was equal to 0.4±0.1 nm (Table S1)

Table S1. Basic topographic parameters of the gold/silica QCM sensors derived from the AFM measurements.

Sensor	Average Surface height between peaks [nm]	*rms* [nm]	Skewness, [1]	Roughness correlation length [nm]	Roughness wavelength [nm]
Gold/Silica	0.4 ±0.1	0.90 ± 0.05	–0.70 ± 0.2	70±10	110± 20

3. Modeling Protein Adsorption Kinetics-the Hybrid RSA Approach

Adsorption kinetics of protein molecules was modeled using the a hybrid approach where the convective–diffusion equation governing the bulk transfer was coupled with the surface boundary layer (SBL) transport equation[4,5]. The available surface function characterizing the mass transfer within the SBL was derived from the coarse-grained, Monte-Carlo type modeling interpolated by a polynomial function expressed in terms of the absolute molecule coverage[6-8]

$$\Theta = S_g \tilde{n} = \left(S_g/m\right) M \tag{S2}$$

where $\tilde{n}$ is the molecule surface concentration, S_g is their characteristic cross-section area, $M = m\tilde{n}$ is the mass coverage per unit area, and m is the mass of a single molecule.

The SBL equation served as the non-linear boundary condition for the bulk mass transfer equation. The boundary value problem formulated in this way was numerically solved by an efficient discretization method [9].

It is worth mentioning that under the regime where the surface transport rate considerably exceeds the bulk transfer rate, which is usually the case for nanoparticle and protein size range and low coverage, the solution of the boundary value problem simplifies to the linear form

$$\Theta = S_g k_c n_b t \tag{S3}$$

where n_b is the number concentration of the particles (molecules) in the bulk, t is the deposition time and k_c is the mass transfer rate constant, which can be calculated for the quasi-impinging jet flow in the QCM cell from the following dependence[5,9]

$$k_c = C_f Q^{1/3} D^{2/3} \tag{S4}$$

where C_f is the parameter depending on the cell geometry, Q is the volumetric flow rate of the protein solution and D is the diffusion coefficient of the molecule.

It is interesting to mention that under this regime bulk transport-controlled regime the mass coverage of particles (molecules) is given by

$$M = k_c c_b t \tag{S5}$$

where $c_b = mn_b$ is their bulk mass concentration.

4. Interpretation of the Quartz Microbalance Results

To determine the sensor impedances, a series of QCM kinetic runs were performed, where the frequency Δf and the dissipation ΔD shifts were registered for first (or third) to 11th overtones (denoted by n_o) as a function of time under various supporting electrolyte concentrations, pHs, suspension flow rates and protein solution concentrations in the bulk. For sake of convenience the frequency shifts were expressed in the normalized form and the dissipation shifts were converted to the bandwidth shifts $-\Delta\Gamma = \frac{1}{2} f_0 \Delta D$, where f_0 is the fundamental frequency of the quartz sensor equal to 5×10^6 Hz. Primary results of these measurements acquired for myoglobin, human serum albumin (HSA) and bovine serum albumin (BSA) are shown in Figure S4 as the dependence of the frequency shifts $-\Delta f/n_o$ (left-hand side (I) - long times, right-hand side (II) - short times) on the adsorption time. Analogous results obtained for fibrinogen are presented in the main manuscript (Figure 3).

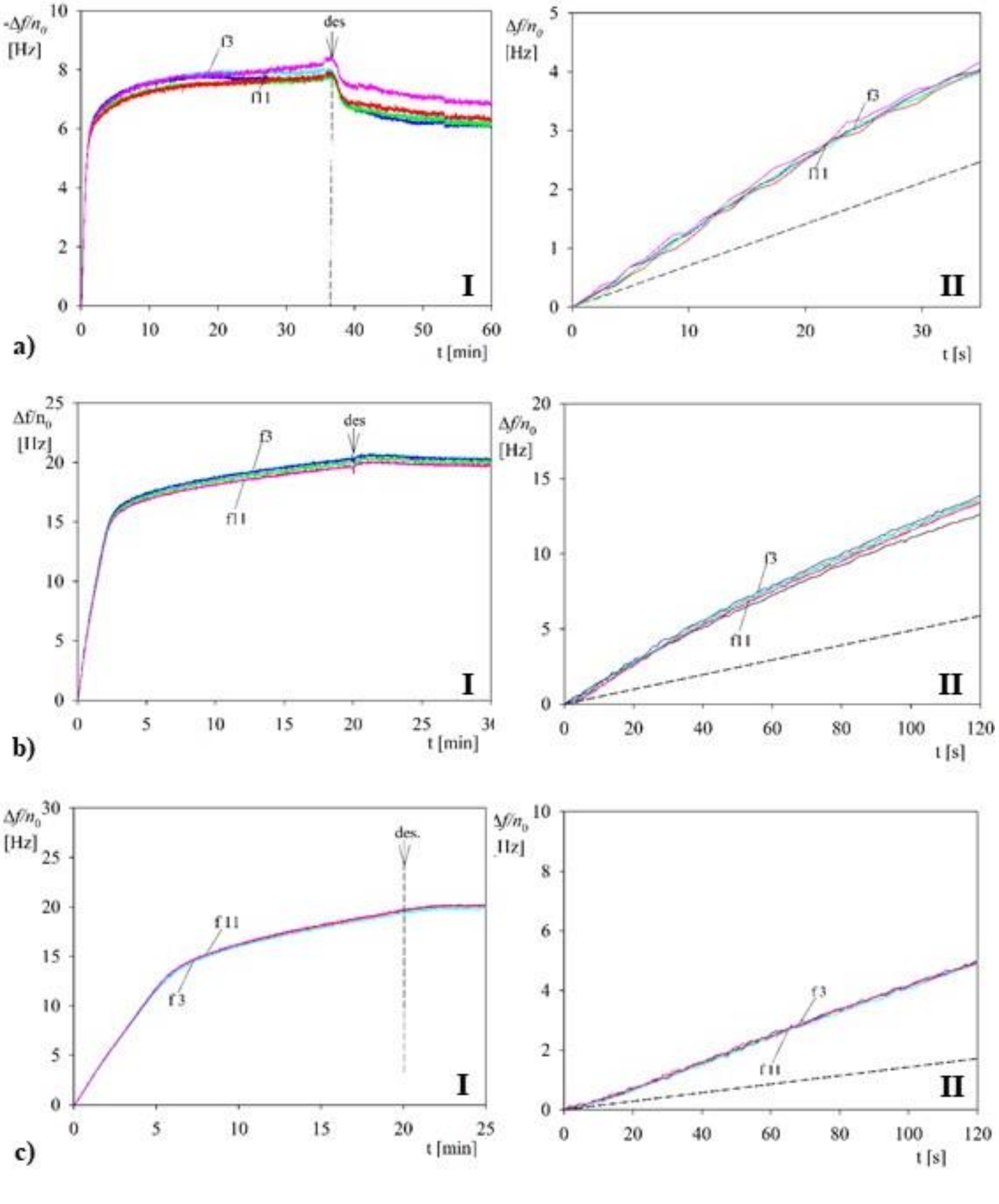

Figure S4. Primary results of QCM measurements acquired for various protein molecules are shown as the dependence of the frequency shifts $-\Delta f/n_o$ (left-hand side (I) - long times, right-hand side (II) - short times) on the adsorption time; silica sensor:
a) myoglobin, pH 3.5, 10 mM NaCl, volumetric flow rate 2.5×10^{-3} cm^3 s^{-1}, bulk concentration 5 mg L^{-1}; b) human serum albumin, pH 3.5, 150 mM NaCl, volumetric flow rate 1.2×10^{-3} cm^3 s^{-1}, bulk concentration 5 mg L^{-1}; c) bovine serum albumin, pH 3.5, 150 mM NaCl, volumetric flow rate 2.5×10^{-3} cm^3 s^{-1}, bulk concentration 10 mg L^{-1} ; The arrows indicate the start of the desorption run where pure electrolyte was flushed through the cell. The dashed lines show the frequency shifts pertinent to the real protein coverage (calculated from the hybrid RSA model).

The dependence of the bandwidth $\Delta\Gamma$ shifts on the adsorption time for myoglobin, human serum albumin (HSA), bovine serum albumin (BSA) and fibrinogen are shown in Figures S5A and S5B.

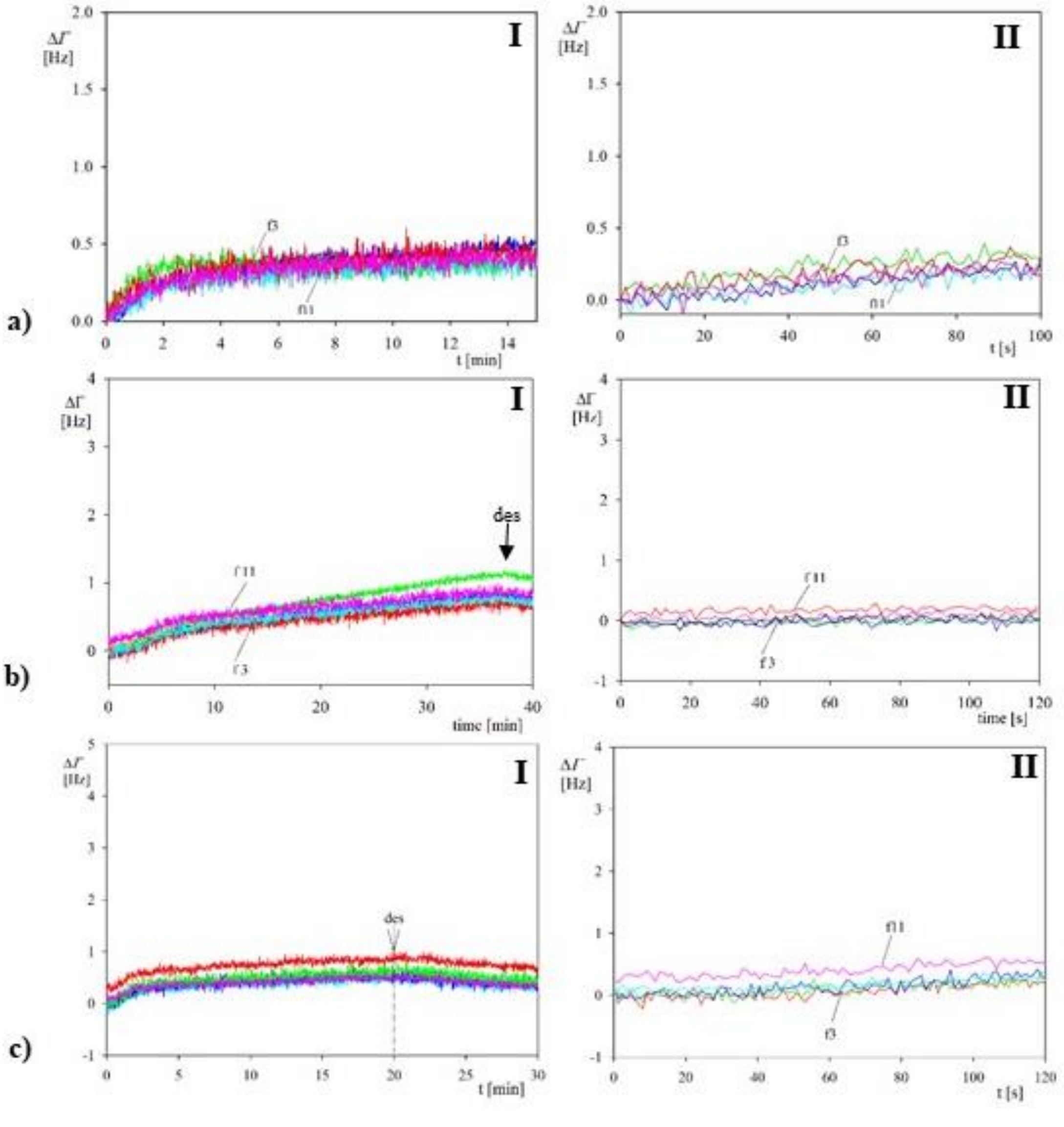

Figure S5A. QCM runs acquired for various protein molecules shown as the dependence of the bandwidth $\Delta\Gamma$ on the adsorption time (I-long time; II- short time):
Part a) myoglobin, pH 3.5, 10 mM NaCl, volumetric flow rate 2.5×10^{-3} cm^3 s^{-1}, bulk concentration 5 mg L^{-1}; Part b) bovine serum albumin, pH 3.5, 150 mM NaCl, volumetric flow rate 1.2×10^{-3} cm^3 s^{-1}, bulk concentration 2 mg L^{-1}; Part c) human serum albumin, pH 3.5, 150 mM NaCl, volumetric flow rate 2.5×10^{-3} cm^3 s^{-1}, bulk concentration 10 mg L^{-1}; The arrows indicate the start of the desorption run, where pure electrolyte was flushed through the cell.

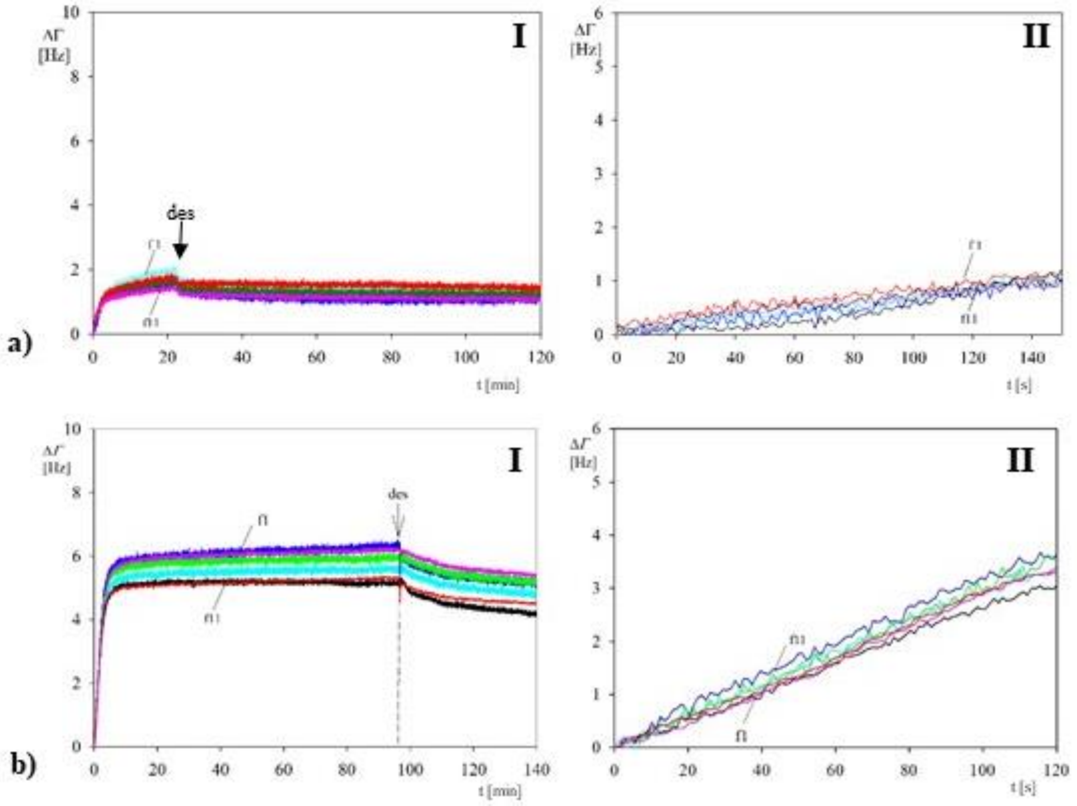

Figure S5B. QCM runs acquired for various protein molecules shown as the dependence of the bandwidth $\Delta\Gamma$ on the adsorption time (I-long time; II- short time)**:** a) fibrinogen, I = 10 mM NaCl, pH 3.5, volumetric flow rate 2.5 x 10^{-3} cm^3 s^{-1}, bulk protein concentration 10 mg L^{-1}; b) fibrinogen, I = 10 mM NaCl, pH 7.4, volumetric flow rate 2.5 x 10^{-3} cm^3 s^{-1}, bulk protein concentration 10 mg L^{-1}. The arrows indicate the start of the desorption run, where pure electrolyte was flushed through the cell.

As can be seen in Figures S5, the $\Delta\Gamma$ signals were smaller than one Hz, even for the maximum adsorption time, only for fibrinogen at pH 7.4 $\Delta\Gamma$ attained 6 Hz for the 1st overtone and the time of 100 min. For comparison, analogous $-\Delta f/n_0$ shifts for the maximum time amounted to ca 20 and 80 Hz, for albumins and fibrinogen, respectively (see Figures S4A, and Figure 3 manuscript), i.e., they were more than one order of magnitude larger. This qualitatively agrees with the theoretically predicted trend (see Figure S2b) and indicates that the dissipation signals are of smaller utility.

Using the $-\Delta f/n_0$ and $\Delta\Gamma$ dependencies derived from experiments, the complex impedance $Z(t)$ components can be calculated as[10-12]

$$\mathrm{Im}[Z(t)] = \frac{\pi Z_q n_o}{f_o}\left[-\frac{\Delta f(t)}{n_o}\right]$$
(S6)
$$\mathrm{Re}[Z(t)] = -\frac{\pi Z_q n_0}{f_0}\Delta\Gamma(t)$$

where Z_q is the acoustic impedance of quartz equal to 8.8 $\times 10^6$ kg m^{-2} Hz^{-1}.

For a purely inertia load, the impedance is given by[11-12]

$$\mathrm{Im}[Z(t)] = \omega m\tilde{n} = \omega M(t)$$
(S7)
$$\mathrm{Re}[Z(t)] = 0$$

where $\omega = 2\pi f_0 n_0$ is the angular velocity of the sensor oscillations, m is the protein molecule mass, $\tilde{n}(t)$ is the surface density of protein molecules, and $M(t)$ is the real mass of the protein layer (referred to as the dry mass), which was obtained from the RSA modeling.

Using this inertia load impedance as a scaling variable, one can calculate the components of the normalized impedance $\bar{Z} = \frac{Z(t)}{2\pi f_0 n_0 M(t)}$ from the following dependences

$$\mathrm{Im}[Z(t)] = \bar{Z}_{im} = \frac{Z_q}{2f_0^2 M(t)}\left[-\frac{\Delta f(t)}{n_0}\right] = \frac{M_Q(t)}{M(t)}$$
(S8)
$$\mathrm{Re}[Z(t)] = \bar{Z}_{re} = -\frac{Z_q}{2f_0^2 M(t)}\Delta\Gamma(t) = -\frac{C_s \Delta\Gamma(t)}{M(t)}$$

where

$$M_Q(t) = -C_s\Delta f(t)/n_0 \quad (S9)$$

is the apparent QCM mass coverage often referred to as the 'wet' mass, and $C_s = \frac{Z_q}{2f_0^2}$ is the Sauerbrey constant equal to 0.177 (mg m^{-2}) Hz^{-1} for $f_0 = 5\times10^6$ Hz.

It is interesting to observe that the $\bar{Z}_{im}$ impedance component has a simple physical interpretation as the ratio of the apparent coverage of the protein layer calculated using the Sauerbrey constant to the real layer coverage.

Using eq. S8, the experimental impedance in the limit of a short time can be determined from the formula

$$\bar{Z}_{im}^{0} = \left(\frac{dM_Q}{dt}\right)_{t\to 0} / \left(\frac{dM}{dt}\right)_{t\to 0} = C_s s_{lQ}(0)/s_l(0)$$

(S10)

$$\bar{Z}_{re}^{0} = -C_s\left(\frac{d\Delta\Gamma}{dt}\right)_{t\to 0} / \left(\frac{dM}{dt}\right)_{t\to 0} = -C_s s_{l\Gamma}(0)/s_l(0)$$

where $s_{lQ}(0)$, $s_l(0)$ and $s_{l\Gamma}(0)$ are the corresponding slopes of the $-\Delta f(t)/n_0$, $M(t)$ and $\Delta\Gamma(t)$, respectively, in the limit of a short time.

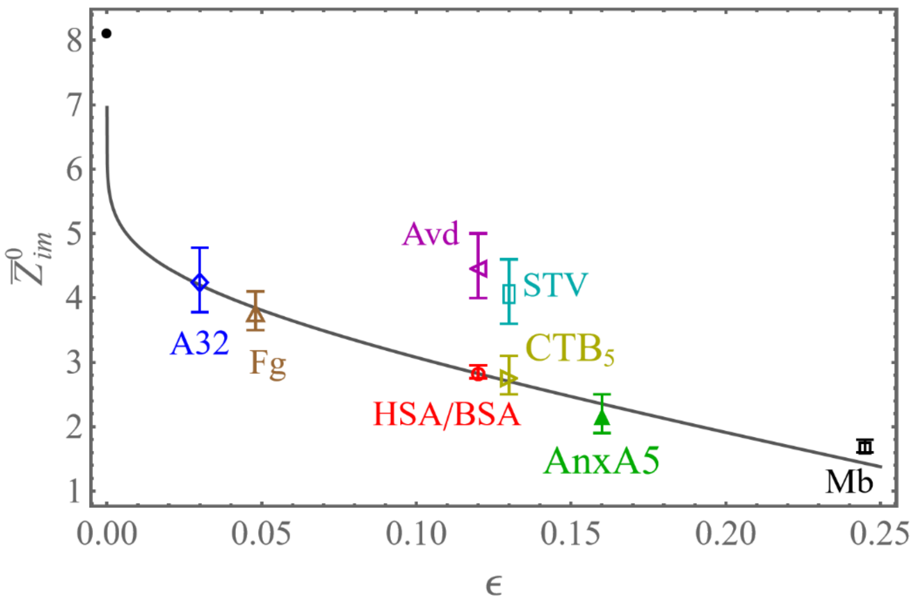

Figure S6. Dependence of $\bar{Z}_{im}^{0}$ on the minimum approach distance $\epsilon = 2h/d_H$ where d_H is the hydrodynamic diameter of the protein. The symbols represent experimental results obtained for various proteins: fibrinogen (Fg), albumins (HSA/BSA), myoglobin (Mb), this work), annexin (AnxA5), the cholera toxin subunit (CTB_5)[13], avidin (Avd) and streptavidin (STV)[14]. The reference result for spherical amidine polymer particles (A32) is also shown. The solid line represents the theoretical results derived from eq 7 (manuscript), and the black dot shows the limiting result predicted for a stiff contact at $\epsilon \approx 0$.

The dependence of the $\bar{Z}_{im}^{0}$ impedance on the scaled minimum distance parameter $\epsilon = 2h/d_H$, where h is the physical minimum distance of 0.4 nm, and d_H is the hydrodynamic diameter of the molecule derived from the DLS measurements of the diffusion coefficient, is presented in Figure S6. As can be seen, the theoretical results derived from eq 7 (manuscript) assuming the lubricated contact of the molecules with the sensor adequately reflect the experimental measurement for most proteins comprising annexin (AnxA5) and the cholera toxin subunit (CTB_5) investigated using simultaneous QCM and ellipsometric measurements[13]. A more significant deviation, exceeding the experimental error, is only observed for avidin (Av) and streptavidin (Sav), where simultaneous QCM and reflectometric measurements were applied [14]. It is also worth mentioning that the results obtained for amidine polymer particles (A32) of a spherical shape and a size of 32 nm agree with the theory.

However, in the general case, the impedance components change with the deposition time, which implies that they also change with the particle coverage $M(t)$. This dependence can be acquired using experimental results if one observes that the adsorption time t_m corresponding to a given mass coverage can be calculated by a numerical inversion of the $M(t)$ function known from the RSA modeling, i.e.,

$$t_m = M^{-1}(t) \tag{S11}$$

where M^{-1} is the inverse of $M(t)$.

Knowing t_m the impedance components can be calculated from eq S8

$$\bar{Z}_{im}(M) = \frac{-C_s \Delta f(t_m)/n_0}{M(t_m)} = \frac{M_Q(t_m)}{M(t_m)}$$

(S12)

$$\bar{Z}_{re}(M) = -C_s \frac{\Delta \Gamma(t_m)}{M(t_m)}$$

References

1. Fouxon, I.; Rubinstein, B.Y.; Leshansky, M.A. Excess shear force exerted on an oscillating plate due to a nearby particle. *Phys. Rev. Fluids*. **2023**, 8, 054104-1-35.
2. Leshansky, A.M.; Rubinstein, B.Y.; Fouxon, I.; Johannsmann, D.; Sadowska, M.; Adamczyk, Z. Quartz crystal microbalance frequency response to discrete adsorbates in liquids. *Anal. Chem*. **2024**, 96, 10559–10568.
3. Adamczyk, Z.; Sadowska, M.; Nattich-Rak, M. Quantifying nanoparticle layer topography: Theoretical modeling and atomic force microscopy investigations. *Langmuir*. **2023**, 39, 15067-15077.
4. Adamczyk, Z. Kinetics of diffusion-controlled adsorption of colloid particles and proteins. *J. Colloid Interface Sci*. **2000**, 229, 477-489.
5. Adamczyk, Z. Particles at interfaces: Interactions, deposition, structure. Elsevier. **2017**.
6. Schaaf, P.; Talbot, J. Surface exclusion effects in adsorption processes. *J. Chem. Phys.* **1989**, 91, 4401–4409.
7. Ricci, S.M.; Talbot, J.; Tarjus, G.; Viot, P. Random sequential adsorption of anisotropic particles. II. Low coverage kinetics. *J. Chem. Phys*. (**1992**), 97, 5219-5228.
8. Talbot, J.; Tarjus, G.; Van Tassel, P.R.; Viot, P. From car parking to protein adsorption: an overview of sequential adsorption processes. *Colloids Surf A Physicochem Eng Asp*. **2000**, 165, 287–324.
9. Adamczyk, Z.; Morga, M.; Nattich-Rak M.; Sadowska M. Nanopartilce and bioparticle deposition kinetics, *Advances Colloid Interface Sci*. **2022**, 302, 102630.
10. Johansmann, D., Reviakine, I.; Richter, R.P. Dissipation in Films of Adsorbed Nanospheres Studied by Quartz Crystal Microbalance (QCM). *Anal. Chem*. **2009**, 81, 8167-8176; https://doi.org/10.1021/ac901381z
11. Meléndez, M.; Vázquez-Quesada, A.; Delgado-Buscalioni, R. Load impedance of immersed layers on the quartz crystal microbalance: A comparison with colloidal suspensions of spheres. *Langmuir*. **2020**, 36, 9225−9234.
12. Delgado-Buscalioni, R. Coverage effects in quartz crystal microbalance measurements with suspended and adsorbed particles. *Langmuir*. **2024**, 40, 580-593.
13. Carton I.; Brisson, A.R.; Richter, R.P. Label-free detection of clustering of membrane-bound proteins. *Anal. Chem*. **2010**, 82, 9275-9281; DOI: 10.1021/ac102495q
14. Bingen, P.; Wang, G.; Steinmetz, N.F.; Rodahl, M.; Richter, R.P. Solvation effects in the quartz crystal microbalance with dissipation monitoring response to biomolecular adsorption. A phenomenological approach. *Anal. Chem*. **2008**, 80, 8880-8890; https://doi.org/10.1021/ac8011686.
